# Insights Into Informal Caregivers’ Well-being: A Longitudinal Analysis of Care Intensity, Care Location, and Care Relationship

**DOI:** 10.1093/geronb/gbad166

**Published:** 2024-01-31

**Authors:** Yanan Zhang, Matthew R Bennett

**Affiliations:** Oxford Institute of Population Ageing, University of Oxford, Oxford, UK; School of Social Policy, University of Birmingham, Edgbaston, UK; (Social Sciences Section)

**Keywords:** Care intensity, Care relationships, Coresident care, Informal care, Psychological well-being

## Abstract

**Objectives:**

This study investigates the psychological well-being of informal caregivers over time. It identifies the thresholds (or “tipping points”) of caring intensity at which caregiving is associated with lower psychological well-being, and how this varies by care location and caregiver–care recipient relationships. It also examines how caring location and relationship are linked to informal caregivers’ psychological well-being while controlling for caring intensity.

**Methods:**

Waves 1–18 (1991–2009) of the harmonized British Household Panel Survey and Waves 1–8 (2009–2017) of the U.K. Household Longitudinal Study were analyzed. Psychological well-being was measured using the General Health Questionnaire (GHQ)-12 score. Care intensity was measured by the weekly hours of care provided. Fixed-effects estimators were applied to the GHQ-12 score of caregivers across different care intensities, caring locations, and caring relationships.

**Results:**

All levels of informal care intensity are associated with lower psychological well-being among spousal caregivers. The thresholds to well-being are 5 hours per week when caring for a parent, and 50 hours per week when caring for a child (with a disability or long-term illness). Caring for “other relatives” or nonrelatives is not negatively associated with psychological well-being. The thresholds are 5 hours per week for both coresident and extraresident caregivers. Extraresident caregivers experience better psychological well-being compared to coresident caregivers, given relatively lower weekly care hours. Caring for primary kin (especially spouses) is linked to lower psychological well-being compared to other caregiving relationships, regardless of care intensity.

**Discussion:**

Policy and practice responses should pay particular attention to spousal caregivers’ well-being. Caregiving relationship has a stronger association with the caregiver’s well-being than care location.

The austerity policies introduced in the wake of the Great Recession substantially reduced the supply of formal long-term care services in the United Kingdom, increasing the importance of the contributions of informal (unpaid) caregivers against a growing demand for care within an aging population (Glasby et al., 2021). Providing informal (unpaid) care to support a family member, friend, neighbor, or others whose needs arise from long-term illness, disability, or advanced age, can positively and negatively affect caregivers’ psychological well-being. This study aims to identify the specific circumstances in which informal caregiving is associated with a shift toward poorer psychological well-being.

Providing informal care is often an act of love or duty, and is linked to many positive benefits, such as a strengthened bond between the caregiver and care recipients, and higher levels of personal satisfaction and purpose in life, stemming from doing what is considered dutiful or right (Litwin et al., 2014). However, all too often providing care comes at a cost to finances, relationships, and health (Keating et al., 2021). The stress process model developed by Pearlin et al. (1990) provides insights into the link between informal care and psychological well-being. Care can involve complex tasks, may feature problem behaviors of the person cared for, involve the deterioration of existing relationships, and may restrict other forms of work and social activities, reducing caregivers’ psychological well-being (Floridi et al., 2022; Verbakel, 2014; Xue et al., 2020; Zhang et al., 2021).

Understanding how and under what circumstances informal caregivers’ psychological well-being is negatively affected is crucial to maintaining the sustainability of the long-term care systems in the United Kingdom (Keating et al., 2021), where an estimated 6.5 million people are informal caregivers (Zhang et al., 2019), and 12,000 people become caregivers daily (Petrillo et al., 2022). The support and care that informal caregivers provided in England and Wales was valued at £162 billion in 2021 in formal care costs—nearly matching the entire National Health Service budget of £164 billion for that year (Petrillo & Bennett, 2023). The health and long-term care system would collapse without the contributions of informal caregivers. However, their contributions and needs frequently remain “invisible,” and adult social care is often low-profile, misunderstood, and commonly overlooked by the public, media, and policymakers (Glasby et al., 2023).

Care location, caregiver–care recipient relationship, and care intensity interact and influence caregivers’ psychological well-being (Bom et al., 2019; Brenna & Di Novi, 2016; Hirst, 2003; Lin et al., 2012; Mentzakis et al., 2009; Penning & Wu, 2015). For example, providing care to a family member at home may involve longer hours and more complex tasks compared to caring for a distant relative in an assisted living facility. However, most studies fail to capture the complex nature of caring. More specifically, they do not disentangle the impact of care location, care relationship, and care intensity—factors that are not experienced in isolation (Andrén & Elmståhl, 2007; Litwin et al., 2014; Robison et al., 2009). The literature examining the impact of care intensity on caregivers’ psychological well-being generally defines levels of care based on thresholds of 10 or 20 hours of care per week (Chen et al., 2019; OECD, 2011; van Den Berg et al., 2014). This warrants further investigation to identify heterogeneities in the thresholds of care intensity across different groups, and how care location and care relationships affect caregivers’ psychological well-being.

In the context of global structural and demographic shifts and the challenges faced by long-term care systems, this study addresses these key gaps in understanding the relationship between the dynamics of informal care and the caregiver’s psychological well-being. Using large-scale nationally representative longitudinal data, it identifies and compares the thresholds of care intensity (“tipping points”) at which care is negatively associated with caregivers’ psychological well-being across care locations and by care relationships. It also contrasts caregivers’ psychological well-being across care locations and by care relationships, while considering the impact of care intensity.

## Theoretical Framework

### Obligation, “Legitimate Excuses”, and Caregivers’ Psychological Well-being

The hierarchical compensatory model proposes an ordered selection process for the arrangement of care, which is normatively defined and reflects the caring obligation between caregivers and care recipients (De Koker, 2008; Lapierre & Keating, 2013; Penning, 1990). This model suggests that individuals first look to those closest to them—usually family members—for care support. If those individuals are unable or unwilling to provide care, they then move down the hierarchy to friends, neighbors, and eventually formal care providers like social workers or healthcare professionals. There are a range of reasons people might use to establish whether they are unable to provide care, including employment, geographical distance, and insufficient skills or competence (Campbell & Martin-Mathews, 2000). “Legitimate excuses” refers to reasons that are generally accepted or recognized as valid for not providing care in certain circumstances. The acceptance of these reasons as “legitimate” is subject to a negotiating process closely linked to care obligations.

Living with someone who has a long-term care need eliminates distance as a reason to refrain from providing care. Caring for a household member is more obligatory compared to looking after someone living separately. Therefore, excuses for not providing (intensive) care will not (easily) be accepted as “legitimate” if a household member needs support (De Koker, 2008). Care provided to a household member is often more intensive than care provided to someone who lives separately in terms of care hours or frequency and types of care tasks that are performed (Campbell & Martin-Matthews, 2000; De Koker, 2008). The coresident caregiver may need to provide support whenever the demand arises. Caregivers have been found to report higher levels of distress and loss of social opportunities when living with a care recipient (Cfas et al., 1998; Hirst, 2003).

Some American and Canadian studies have categorized normative hierarchies of obligation to provide care based on relationship type (Cantor, 1979; Penning, 1990). In those hierarchies, relationships that rank higher have fewer “legitimate excuses” for avoiding the provision of (intensive) care. According to this hierarchical model, when an older adult needs support, normative relationship obligations are ranked in the following order: (1) spouse, (2) children, (3) other relatives (e.g., siblings, nieces, nephews, and grandchildren), (4) nonkin relationships (e.g., friends and neighbors), and finally (5) formal organizations (Lapierre & Keating, 2013; Penning, 1990). Caring relationships with lower levels of normative obligation are associated with a higher degree of voluntary effort (such as nonkin), compared to relationships higher up in the hierarchy (Marks et al., 2002). This greater voluntary effort can contribute to higher levels of personal satisfaction and purpose in life, improving the caregiver’s psychological well-being.

Across the four nations of the United Kingdom, local councils, trusts, or authorities are legally obligated to support individuals with care needs, as well as their informal caregivers, based on varying needs assessment and financial eligibility criteria. However, the resource constraints of these authorities often result in the bulk of care falling upon families, friends, and neighbors (Humphries, 2022). Caring for someone with a long-term illness, disability, or advanced age is often seen as a familial responsibility, particularly, in the context of spousal and parent–child relationships (Pickard et al., 2007). There is an increasing recognition of the role of friends, neighbors, and community networks in providing care, under the background of societal changes such as smaller family size, increased divorce rates, and greater geographic mobility (Zhang, 2023). Policy changes focused on improving formal care arrangements and emphasizing “ageing in place” have implicitly increased the emphasis of informal care “through the back door” with implications on the finances, health, and relationships of informal caregivers (Kodate & Timonen, 2017).

The limited number of studies that examine the association between caring relationships and caregivers’ psychological well-being report inconsistent findings (Robison et al., 2009). Some studies find that among caregivers of patients with dementia, adult children report a higher level of strain than spouses and other family members (Chappell et al., 2014). Adult children who care for older parents may also juggle work and have their own family responsibilities, and these competing commitments are associated with lower well-being (Lin et al., 2012).

Conversely, other research suggests that the caregivers of spouses experience higher stress (Hong & Kim, 2008), more depressive symptoms, and lower subjective well-being than caregivers of parents (Pinquart & Sorensen, 2011). Partners with long-term care needs may not be able to contribute to childcare, household work, or family income, and thus their spousal caregivers have to fully undertake these responsibilities in addition to supporting them. Caring for a spouse or a child is found to be more stressful than providing care for other relationships (Litwin, 2014; Penning & Wu, 2015; Shahly et al., 2013). Parents may have to look after their children with long-term care needs for the rest of their lives. Furthermore, some studies find that caregivers’ well-being is not associated with the care relationship (Robison et al., 2009).

### Caring Intensity and Caregivers’ Psychological Well-being

Care intensity encompasses many aspects, including time commitment, physical strain, emotional demands, and complexity of care tasks. However, measuring these dimensions can be challenging, and in quantitative studies, care intensity is predominately represented by the number of care hours provided (Brenna & Di Novi, 2016; Hirst, 2003). In this study, the term “care intensity” refers to the number of care hours provided, unless otherwise specified.

As illustrated in the stress process model, the care recipients’ cognitive function, comorbidity, physical limitations, and mental health determine the need for care (Swinkels et al., 2019). More hours of care are associated with greater physical and emotional stress. Additionally, time spent on caring crowds out activities that are enjoyable and beneficial for well-being (Verbakel et al., 2018). Care intensity has been recognized as an important determinant of the likelihood of experiencing mental health problems (Bom et al., 2019); caregivers’ psychological distress and strain increase with the number of care hours (Brenna & Di Novi, 2016).

A recent report reveals that people who provide low-intensity care (less than 10 hours per week or between 10 and 20 hours per week) do not necessarily have a higher prevalence of mental health problems compared with noncaregivers in the Organisation for Economic Co-operation and Development (OECD) countries (OECD, 2011). However, caring for more than 20 hours per week is associated with a 20% higher likelihood of experiencing mental health problems. So far, no study systematically examines the thresholds at which care intensity negatively affects caregivers’ psychological well-being. Previous studies often applied 10 or 20 hours per week as a threshold irrespective of care circumstances (Chen et al., 2019; OECD, 2011; van Den Berg et al., 2014). Further analyses are needed to explore psychological well-being across varying intensity thresholds and diverse caregiving groups. Such investigation is vital for identifying caregivers whose well-being is at risk and guiding policy interventions and responses accordingly.

Care intensity is closely linked to care location and caregiver–care recipient relationships. Caregivers who reside with their care recipients often have greater caregiving responsibilities compared to those who live separately (Mentazakis et al., 2009). It’s noteworthy that such living arrangements are more commonly observed among caregivers of spouses and children, as opposed to those in other caregiving relationships. However, most existing studies that examine the impact of caring location and caregiver–care recipient relationships on caregivers’ psychological well-being do not differentiate these factors from caring intensity (Andrén & Elmståhl, 2007; Litwin et al., 2014; Robison et al., 2009). Thus, disentangling the psychological well-being effects of caring intensity improves our understanding of the influence of care locations and of caregiver–care recipient relationships.

### Present Study

This study examines the psychological well-being of informal caregivers by caring intensity, across locations of care, and by different caregiver–care recipient relationships to address two research questions: (1) At what threshold is caring intensity associated with lower psychological well-being for caregivers, across locations of care, and in different caregiver–care recipient relationships? (2) How are care location and caregiver–care recipient relationship linked to caregivers’ psychological well-being at different levels of care intensity?

Following the theoretical framework (obligation and “legitimate excuses”), care location and care relationship potentially influence the caregiver’s psychological well-being via care intensity. Caregivers higher up in the obligation hierarchy tend to have more care responsibilities and fewer “legitimate excuses” for avoiding providing care. They are more likely to provide the types of support that require greater commitment and are associated with more emotional and physical stress. For people lower in the hierarchy of care obligation, providing care is linked to a higher level of voluntary effort and therefore, a greater purpose in life and personal satisfaction, which might be beneficial to well-being. This study hypothesizes that the care hour threshold at which care is associated with lower psychological well-being will be lower for caregivers higher up in the care obligation hierarchy than it is for caregivers lower down in the hierarchy (*H1*). Given the same level of care intensity, caregivers higher up in the hierarchy will have lower psychological well-being compared to caregivers lower down in the hierarchy (*H2*).

## Data and Methods

### Data

Nationally representative longitudinal data are used from Waves 1–18 (1991–2009) of the harmonized British Household Panel Survey (BHPS) and Waves 1–8 (2009–2017) of the U.K. Household Longitudinal Study (UKHLS). More details on the data set can be found in Appendix I (see also University of Essex, 2020). Across all linked waves, there are 611,145 observations for 105,031 individuals aged 16 and over. The surveys ask participants whether they care for a coresident or someone who lives in a separate household, and their relationship with the care recipient. The relationship between respondents and their coresident care recipient is categorized into 30 classifications. However, the data only provide six categories for the relationships between the respondents and their care recipients who live separately (see more details in Appendix II). The data do not reveal if the care recipient who lives outside the household is the caregiver’s spouse, child, or “other relative.” For this reason, when exploring the association between care relationships and caregivers’ well-being, the sample is restricted to noncaregivers and coresident caregivers (*n* = 431,817). To isolate the influence of each caring relationship, the sample excludes those who care for multiple relationships (*n* = 314).

To investigate the role of care location, respondents who live alone are excluded (*n* = 89,804). It is well-established that there is a significant difference in mental health between those who live alone and those who live with others, especially among older adults (Smith & Victor, 2019). People who live alone can only look after someone who lives outside the household, and the negative psychological well-being effects of living alone may lower the extraresident caregivers’ mental health. Respondents who provide care across more than one location are also excluded (*n* = 5,293). Cases with missing values for the dependent and key explanatory variables are dropped (approximately 7% of the sample have missing observations—the majority of them are for General Health Questionnaire [GHQ]-12 and income variables), resulting in a final analytical sample of 324,240–369,053 observations. All the analyses are conducted with Stata 15.

### Measurement of Care

Respondents are defined as informal caregivers if they provide regular support or help for someone who is sick, disabled, or older adults (see Appendix II for more information). The data provide time intervals for the number of hours per week that respondents spent caring. Making use of the information, six dummy variables were created capturing care intensity: *CareInt(0*–*4 hr)*, *CareInt(5*–*9 hr)*, *CareInt(10*–*19 hr)*, *CareInt(20*–*34 hr)*, *CareInt(35*–*49 hr)*, and *CareInt(50+ hr)*.

To differentiate care locations, caregivers are categorized into two groups: the subsample of people who care for a household member; and a group who look after someone living separately. Two dummy variables were created to measure caring location: *coresidence* and *extraresidence*. Caregivers were also divided into five groups based on their relationship with the care recipient: the subsample of people who care for a spouse, the group of people who look after a child, caregivers of a parent, caregivers of “other relatives,” and caregivers of nonrelatives. Correspondingly, five dummy variables were created to indicate each group: *Spouse*, *Child*, *Parent*, *OtherRelative*, and *NonRelative.*

### Measurement of Psychological Well-being

Psychological well-being is measured using the 12-item scaled version of the GHQ-12. The GHQ is a screening tool to detect emotional or mental distress and examines the risk of having a psychiatric disorder, which has been validated among the general population in the United Kingdom (Hankins, 2008; Pevalin, 2000). The Likert scoring of the GHQ-12 is discrete and ranges from 0 to 36. A higher GHQ-12 score represents more depressive symptoms and lower psychological well-being.

### Model

Following OECD (2011), Carmichael et al. (2010), and King and Pickard (2013), the baseline model below is used to identify the care hour threshold at which caregiving is associated with lower psychological well-being:


$$\begin{array}{l} \begin{array}{lllll} GHQ_{it} & =\alpha_{0}+\;\alpha_{1}CareInt{\left(0-4\;hr\right)}_{it}\; & +\;\alpha_{2}CareInt{\left(5-9\;hr\right)}_{it}+\;\alpha_{3}CareInt{\left(10-19\;hr\right)}_{it}\; & +\;\alpha_{4}CareInt{\left(20-34\;hr\right)}_{it}+\;\alpha_{5}CareInt{\left(35-49\;hr\right)}_{it}\; & +\;\;\alpha_{6}CareInt{\left(50+hr\right)}_{it}\;\;+X_{it}^{\prime }\beta +\epsilon_{i}+\;v_{t}+u_{it}\;\;\;\;\;\;\;\;\;\;\;\;\;\;\;\;\;\;\;\;\;\;\;\;\;\;\;\;\;\;\;\;\;\;\;\;\;\;\;\;\;\;\;\;\;\;\;\;\;\;\;\;\;\;\;\;\;\;\;\;\;\;\;\;\;\;\;\;\;\;\;\;\;\;\;\;\;\;\;\;\;\;\;\;\;\;\;\;\;\;\;\;\;\;\;\;\;\;\;\;\;\;\;\;\;\;\;\;\;\;\;\;\;\;\;\;\;\;\;\;\;\;\;\;\;\;\;\;\;\;\;\;\;\;\;\;\;\;\;\;\;\;\;\;\;\;\;\;\;\;\;\;\;\;\;\;\;\;\;\;\;\;\;\;\;\;\;\;\;\;\;\;\;\;\;\;\;\;\;\;\;\;\;\;\;\;\;\;\;\;\;\;\;\;\;\;\;\;\;\;\;\;\;\;\;\;\;\;\;\;\;\;\;\;\;\;\;\;\;\;\;\;\;\;\;\;\;\;\;\;\;\;\; \end{array} \end{array}$$ (1)

$GHQ_{it}$ represents the GHQ-12 Likert score for individual *i* in year *t*. $CareInt{\left(0-4\;\;hr\right)}_{it}$ to $CareInt{\left(50+\;hr\right)}_{it}$ are dummy variables for care intensity equal to 1 if individual *i* spends the corresponding hours per week (shown in the parentheses) on caring for someone in year *t*, and 0 otherwise. The reference group is noncaregivers. $\alpha_{1}$–$\alpha_{6}\;$ indicates the difference in the subjective well-being between noncaregivers and those caring for corresponding hours. The threshold of care intensity is defined as the lower bound of the care time interval at which caregivers start to experience lower psychological well-being compared to noncaregivers (a positive and significant α). $X_{it}^{\prime }$ is the vector of control variables that have already been found to influence psychological well-being (Penning & Wu, 2015). It includes educational attainment, age, marital status, disability, financial situation, employment status, and household composition. The definitions of all variables can be found in Appendix III. $\epsilon_{i}$ and $v_{t}$ are the individual-specific time-invariant effects and the business cycle effects. $u_{it}$ is an idiosyncratic error term. The equation is estimated for seven caregiver subsamples: two for caring locations (“within” or “outside” their household) and five for different caregiver–care recipient relationships (spouse, parents, child, “other relative”, and nonrelative).

The following baseline model is used to compare the psychological well-being of informal caregivers by locations of caring:


$$\begin{array}{lll} GHQ_{it}= & \alpha_{0}+\;\alpha_{1}ExtraResidence_{it}+\;\alpha_{2}CoResidence_{it}\;\; & +X_{it}^{\prime }\beta +\epsilon_{i}+v_{t}+u_{it} \end{array}$$ (2)

$ExtraResidence_{it}\;(CoResidence_{it})$ is equal to 1 if individual *i* cares for someone outside (within) the household in year *t*, and zero otherwise. Noncaregivers are the reference group. The equation for comparing the psychological well-being of caregivers by caregiver–care recipient relationship is as follows:


$$\begin{array}{l} \begin{array}{llll} GHQ_{it} & =\alpha_{0}+\;\alpha_{1}Spouse_{it}+\;\alpha_{2}Child_{it}\; & +\alpha_{3}Parent_{it}+\alpha_{4}OtherRelative_{it}\;\;\;\;\;\;\;\;\;\;\;\;\;\;\;\;\;\;\;\;\;\;\;\;\;\;\;\;\;\;\;\;\;\;\;\;\; & +\alpha_{5}Nonrelative_{it}+X_{it}^{\prime }\beta +\epsilon_{i}+v_{t}+u_{it}\; \end{array} \end{array}$$ (3)

$Child_{it}$ $(Parent_{it})$ is equal to 1 if individual *i* looks after his/her child with a disability or long-term illness (parent) in year *i*, and zero otherwise. $Spouse_{it}$, $OtherRelative_{it}$ and $Nonrelative_{it}$ are defined similarly. To disentangle the effects of caring intensity, caregivers are grouped by weekly hours of care: less than 10, 10–19, 20–49, and 50+ hr. To control for the unobserved time-invariant individual characteristics, fixed-effect estimators are applied to these models. The full sample includes 411,885 observations for 80,384 individuals, accounting for noncaregivers, coresident and extraresident caregivers. Of these, 25,627 individuals experienced transitions between different care statuses over time, contributing to a total of 187,269 observations. The considerable variance among individuals ensures that the fixed-effects model has ample information for robust estimation.

## Results

### Descriptive Analysis

**Figure 1.**
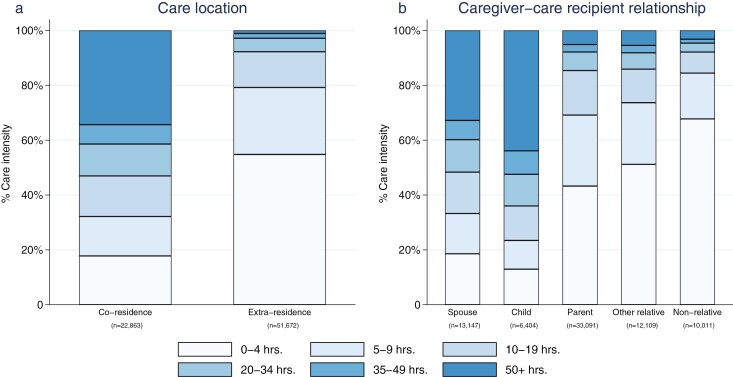
Care intensity by care location and caregiver–care recipient relationship.

Informal caregivers care for more hours if they live with the care recipient (Figure 1). More than 34% of coresident caregivers care for 50+ hours per week; the percentage of caring at this intensity is approximately 1% for those who care for someone outside the household. In contrast, 54.8% of informal caregivers providing care outside the household spend less than 5 hours per week caring; a far higher percentage than those supporting a coresident household member (17.8%). Differentiating informal caregivers by their relationship with the care recipient, the largest proportion of caregivers providing care for 50+ hours per week is among those who look after a child with a disability or long-term illness (43.9%), followed by those who care for a spouse (32.8%). Approximately 70% of those who look after their parents care for less than 10 hours per week; the percentages are 79% and 85% for those who care for an “other relative” and a nonrelative, respectively.

Descriptive statistics (Table 1) show that compared with those who care for someone living in a separate household, living with the person cared for is associated with lower psychological well-being. The average GHQ-12 score (arithmetic mean) is 12.266 for coresident informal caregivers and 11.277 for extraresident informal caregivers (a lower GHQ-12 score refers to fewer depressive symptoms and higher psychological well-being, and vice versa). In contrast, noncaregivers have better psychological well-being, with an average GHQ-12 score of 10.856. Informal caregivers of a spouse or child report higher mean GHQ-12 scores (12.323 and 13.130, respectively). Caregivers who look after a nonrelative have a GHQ-12 score similar to those of noncaregivers. The average age of caregivers of a spouse is 62 years old, compared with lower ages for those who care for their child, parent, “other relative,” or nonrelative (46, 48, 44, and 54 years old, respectively). Noncaregivers are generally younger, tend to be engaged in paid work, and are less likely to be disabled or retired.

**Table 1.** Descriptive Statistics of the Variables

Variable	All	Noncaregiver^a^	Care location^b^		Caregiver–care recipient relationship^c^				
			Coresidence	Extraresidence	Spouse	Child	Parent	Other relative	Nonrelative
GHQ-12	10.987	10.856****	12.266	11.277****	12.323	13.130****	11.602****	11.409***	10.898****
	(5.346)	(5.283)	(5.909)	(5.404)	(5.739)	(6.374)	(5.563)	(5.603)	(5.291)
Degree	0.197	0.205****	0.117	0.177****	0.086	0.1620****	0.1810****	0.1442****	0.162****
	(0.398)	(0.404)	(0.322)	(0.382)	(0.280)	(0.368)	(0.385)	(0.351)	(0.368)
Age	45.166	43.751****	52.365	51.216****	62.032	45.720****	48.436****	44.076****	54.169***
	(17.165)	(17.111)	(18.068)	(14.830)	(14.477)	(12.495)	(12.183)	(18.609)	(16.259)
Married	0.852	0.862****	0.864	0.778****	0.997	0.816****	0.886****	0.839****	0.855****
	(0.356)	(0.345)	(0.343)	(0.415)	(0.051)	(0.388)	(0.319)	(0.368)	(0.352)
Widowed	0.022	0.016****	0.018	0.064****	0.000	0.044****	0.010****	0.018****	0.0240****
	(0.148)	(0.126)	(0.134)	(0.245)	(0.009)	(0.206)	(0.098)	(0.132)	(0.153)
Disabled	0.213	0.198****	0.342	0.260****	0.404	0.347****	0.250****	0.253****	0.265****
	(0.410)	(0.398)	(0.474)	(0.439)	(0.491)	(0.476)	(0.433)	(0.435)	(0.441)
Income	7.094	7.103***	6.900	7.124****	6.845	7.208****	7.125****	6.9172****	6.962****
	(1.087)	(1.105)	(0.954)	(1.013)	(0.868)	(0.872)	(1.060)	(1.087)	(1.043)
House ownership	0.753	0.755	0.654	0.785****	0.672	0.578****	0.803****	0.734****	0.791****
	(0.431)	(0.430)	(0.476)	(0.411)	(0.470)	(0.494)	(0.398)	(0.442)	(0.407)
Household size	3.111	3.177****	3.236	2.625****	2.563	4.063****	3.124****	3.194****	2.775****
	(1.297)	(1.267)	(1.498)	(1.289)	(1.073)	(1.310)	(1.276)	(1.361)	(1.129)
Work	0.622	0.643****	0.373	0.596****	0.252	0.449****	0.670****	0.545****	0.469****
	(0.485)	(0.479)	(0.484)	(0.491)	(0.434)	(0.497)	(0.470)	(0.498)	(0.499)
Retired	0.183	0.163****	0.322	0.248****	0.532	0.103****	0.139****	0.213***	0.363****
	(0.386)	(0.369)	(0.467)	(0.432)	(0.499)	(0.304)	(0.346)	(0.410)	(0.481)
Observation	411,885	337,341	22,863	51,672	13,147	6,404	33,091	12,109	10,011

*Notes*: Mean and standard deviation (in parentheses) are reported, and effective sample sizes are used. GHQ-12 = General Health Questionnaire.

^a^Mean-comparison tests between caregiver and noncaregivers are conducted.

^b^Mean-comparison tests between the caregiver groups of Coresidence and Extraresidence are conducted.

^c^Mean-comparison tests with spousal caregiver are conducted.

****p < .0001.

***p < .001.

**p < .01.

*p < .05.

### Multivariate Analysis

#### Caring intensity and caregivers’ psychological well-being by care location

Table 2 shows the relationship between caring intensity and psychological well-being (depressive symptoms) by caring location. Column 1 indicates the difference in the GHQ-12 score between noncaregivers and coresident caregivers who care for 0–4 hours per week (β = 0.146, 95% CI: −0.008 to 0.299) is not statistically significant. In contrast, caring for 0–4 hours per week is associated with a 0.132 lower GHQ-12 score (95% CI: −0.196 to −0.069) among extraresident caregivers. Any caring intensity higher than 5 hours per week is associated with more depressive symptoms for both coresident and extraresident caregivers. For instance, those caring for 5–9 hours per week have a 0.262 higher GHQ-12 score (95% CI: 0.092 to 0.433) than noncaregivers, if they live with a care recipient. The results also show that higher intensities of care are associated with larger differences in depressive symptoms between caregivers and noncaregivers: as shown in Column 2, 0.102 for 5–9 hours per week (95% CI: 0.013 to 0.191), 0.294 for 10–19 hours per week (95% CI: 0.174 to 0.415), 0.541 for 20–34 hours per week (95% CI: 0.394 to 0.734), 0.573 for 35–49 hours per week (95% CI: 0.252 to 0.895), and 0.841 for 50+ hours per week (95% CI: 0.434 to 1.249) for extraresident caregivers.

**Table 2.** Psychological Well-being and Caring Intensity by Location of Care Provision

Variables	(1)	(2)
	Coresidence	Extraresidence
	β (95% CI)	β (95% CI)
0–4 hr	0.146	−0.132***
	(−0.008 to 0.299)	(−0.196 to 0.069)
5–9 hr	0.262**	0.102*
	(0.092 to 0.433)	(0.013 to 0.191)
10–19 hr	0.398***	0.294***
	(0.228 to 0.568)	(0.174 to 0.415)
20–34 hr	0.617***	0.541***
	(0.425 to 0.809)	(0.349 to 0.734)
35–49 hr	0.528***	0.573***
	(0.284 to 0.772)	(0.252 to 0.895)
50+ hr	0.949***	0.841***
	(0.805 to 1.094)	(0.434 to 1.249)
Degree	0.605***	0.551***
	(0.469 to 0.742)	(0.420 to 0.683)
Age	0.013	0.003
	(−0.042 to 0.068)	(−0.049 to 0.056)
Married	0.088*	0.056
	(0.008 to 0.167)	(−0.019 to 0.131)
Widowed	1.243***	1.146***
	(0.970 to 1.516)	(0.909 to 1.384)
Disabled	0.901***	0.870***
	(0.847 to 0.956)	(0.819 to 0.922)
Income	0.063	0.071
	(−0.023 to 0.149)	(−0.011 to 0.153)
Income square	−0.004	−0.004
	(−0.011 to 0.004)	(−0.011 to 0.004)
House ownership	−0.036	−0.016
	(−0.114 to 0.043)	(−0.092 to 0.060)
Household size	0.038**	0.043***
	(0.014 to 0.062)	(0.020 to 0.066)
Work	−0.868***	−0.894***
	(−0.931 to −0.805)	(−0.955 to −0.833)
Retired	−1.368***	−1.458***
	(−1.461 to −1.275)	(−1.546 to −1.370)
Constant	9.874***	10.147***
	(8.286 to 11.462)	(8.610 to 11.683)
Observation	360,204	389,013
Number of individuals	75,705	77,707

*Notes*: A fixed-effects model is applied. 95% confidence interval is given in parentheses. Year dummies are included in all models, but their coefficients are not reported, for brevity.

***p < .001.

**p < .01.

*p < .05.

#### Caring intensity and caregivers’ psychological well-being by care relationship

Table 3 shows the link between caring intensity and psychological well-being (depressive symptoms) for each caregiver–care recipient relationship. Column 1 shows that spousal caregivers report a higher GHQ-12 score than noncaregivers, regardless of the level of care intensity. People who spend 50+ hours per week caring for a spouse have a 1.273 higher GHQ-12 score (95% CI: 1.088 to 1.459) than noncaregivers. There is no statistically significant difference in the GHQ-12 score between noncaregivers and those who look after their child for less than 50 hours per week (Column 2). Parents who spend more than 50 hours per week taking care of their child with a long-term illness or disability report a 0.692 higher GHQ-12 score than noncaregivers (95% CI: 0.434 to 0.950).

**Table 3.** Psychological Well-being and Caring Intensity by Caregiver–Care Recipient Relationship

Variables	(1)	(2)	(3)	(4)	(5)
	Spouse	Child	Parent	Other relative	Nonrelative
	β (95% CI)	β (95% CI)	β (95% CI)	β (95% CI)	β (95% CI)
0–4 hr	0.396***	0.037	−0.071	−0.180**	−0.183**
	(0.202 to 0.590)	(−0.300 to 0.373)	(−0.156 to 0.014)	(−0.308 to −0.053)	(−0.303 to −0.063)
5–9 hr	0.386***	0.089	0.201***	0.061	−0.210
	(0.168 to 0.604)	(−0.279 to 0.458)	(0.095 to 0.307)	(−0.127 to 0.249)	(−0.437 to 0.017)
10–19 hr	0.588***	0.288	0.377***	0.118	−0.289
	(0.370 to 0.805)	(−0.054 to 0.629)	(0.243 to 0.511)	(−0.138 to 0.374)	(−0.626 to 0.048)
20–34 hr	0.955***	0.322	0.641***	0.139	−0.286
	(0.708 to 1.202)	(−0.041 to 0.685)	(0.438 to 0.844)	(−0.234 to 0.512)	(−0.820 to 0.248)
35–49 hr	0.967***	0.188	0.351*	0.483	−0.065
	(0.652 to 1.283)	(−0.237 to 0.612)	(0.032 to 0.670)	(−0.098 to 1.064)	(−0.915 to 0.785)
50+ hr	1.273***	0.692***	0.692***	0.189	−0.446
	(1.088 to 1.459)	(0.434 to 0.950)	(0.421 to 0.963)	(−0.278 to 0.657)	(−1.093 to 0.201)
Degree	0.561***	0.569***	0.564***	0.553***	0.569***
	(0.423 to 0.699)	(0.431 to 0.708)	(0.430 to 0.699)	(0.418 to 0.689)	(0.433 to 0.706)
Age	0.003	0.009	0.008	0.022	0.009
	(−0.053 to 0.059)	(−0.047 to 0.066)	(−0.047 to 0.062)	(−0.034 to 0.078)	(−0.047 to 0.065)
Married	0.056	0.045	0.071	0.053	0.064
	(−0.025 to 0.138)	(−0.036 to 0.126)	(−0.007 to 0.149)	(−0.027 to 0.133)	(−0.017 to 0.144)
Widowed	1.286***	1.254***	1.378***	1.231***	1.388***
	(1.000 to 1.572)	(0.960 to 1.548)	(1.092 to 1.663)	(0.936 to 1.526)	(1.093 to 1.683)
Disabled	0.888***	0.899***	0.882***	0.886***	0.881***
	(0.833 to 0.943)	(0.843 to 0.955)	(0.829 to 0.935)	(0.831 to 0.942)	(0.826 to 0.937)
Income	0.057	0.054	0.074	0.056	0.054
	(−0.030 to 0.143)	(−0.033 to 0.141)	(−0.009 to 0.158)	(−0.030 to 0.141)	(−0.032 to 0.140)
Income square	−0.003	−0.003	−0.004	−0.003	−0.003
	(−0.011 to 0.005)	(−0.011 to 0.005)	(−0.011 to 0.003)	(−0.010 to 0.005)	(−0.010 to 0.005)
House ownership	−0.034	−0.039	−0.033	−0.042	−0.041
	(−0.113 to 0.045)	(−0.119 to 0.041)	(−0.111 to 0.045)	(−0.121 to 0.037)	(−0.121 to 0.038)
Household size	0.045***	0.037**	0.040***	0.038**	0.051***
	(0.020 to 0.069)	(0.013 to 0.062)	(0.017 to 0.064)	(0.014 to 0.062)	(0.026 to 0.075)
Work	−0.864***	−0.865***	−0.889***	−0.850***	−0.863***
	(−0.928 to −0.799)	(−0.930 to −0.800)	(−0.951 to −0.826)	(−0.914 to −0.785)	(−0.927 to −0.798)
Retired	−1.366***	−1.421***	−1.462***	−1.384***	−1.396***
	(−1.459 to −1.272)	(−1.518 to −1.324)	(−1.554 to −1.371)	(−1.480 to −1.288)	(−1.492 to −1.301)
Constant	10.154***	10.019***	9.994***	9.620***	9.962***
	(8.539 to 11.768)	(8.417 to 11.621)	(8.446 to 11.541)	(8.037 to 11.204)	(8.358 to 11.565)
Observation	350,488	343,745	370,432	349,450	347,352
Number of individuals	74,276	73,461	75,298	74,113	73,520

*Notes*: A fixed-effects model is applied. 95% confidence interval is given in parentheses. Year dummies are included in all models, but their coefficients are not reported, for brevity.

***p < .001.

**p < .01.

*p < .05.

People who spend 0–4 hours per week caring for their parents have a similar GHQ-12 score as noncaregivers (β = −0.071, 95% CI: −0.156 to 0.014). However, if the care is given for more than 5 hours per week, those who look after their parent have a higher GHQ-12 score than noncaregivers. Caring for 0–4 hours per week is associated with a 0.180 lower GHQ-12 score for those caring for “other relatives” (95% CI: −0.308 to −0.053) and a 0.183 lower GHQ-12 score for respondents who look after nonrelatives (95% CI: −0.303 to −0.063). There is no significant difference in the depressive symptoms of noncaregivers and respondents who spend over 5 hours per week caring for “other relatives” or nonrelatives.

#### Psychological well-being of caregivers by care location, care relationship, and caring intensity

**Figure 2.**
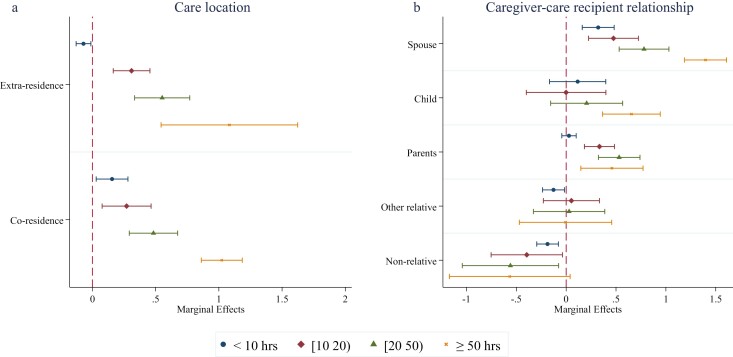
Psychological well-being and caring by location, caregiver–care recipient relationship, and care intensity. The error bars indicate the 95% confidence interval. A fixed-effects model is applied, which controls for educational attainment, age, marital status, disability, financial and employment status, and year dummies. Full results are given in Appendix Tables 2 and 3 in Appendix IV.

Figure 2 compares caregivers’ psychological well-being (depressive symptoms) by caring location and by caregiver–care recipient relationship, differentiating subsamples by caring intensity. Figure 2A shows that coresident caregivers report lower psychological well-being (more depressive symptoms) compared to extraresident caregivers, but only if they care for fewer than 10 hours per week. Given the care intensity of less than 10 hours per week, Figure 2B shows caregivers’ GHQ-12 score is 0.321 higher than noncaregivers when care is given to a spouse (95% CI: 0.161 to 0.481). At the same care intensity, caring for “other relatives” and nonrelatives is associated with a lower GHQ-12 score (−0.127 [95% CI: −0.237 to −0.017] and −0.187 [95% CI: −0.295 to −0.078] respectively); there is no statistically significant difference in the psychological well-being between noncaregivers and caregivers of a child, nor a difference between noncaregivers and caregivers of a parent.

For the group caring between 10 and 19 hours per week, caring for a spouse or parent is linked to lower psychological well-being than noncaregivers (0.475 [95% CI: 0.224 to 0.725] and 0.334 [95% CI: 0.183 to 0.485], respectively). There is no statistically significant difference between the GHQ-12 scores of noncaregivers and caregivers for children, nor between noncaregivers and caregivers for “other relatives.” Caring for nonrelatives is associated with a 0.395 lower GHQ-12 score (95% CI: −0.752 to −0.037). Similar results are found for people who care for 20–49 hours per week. When care is given at the highest intensity level (≥50 hours per week), caring for a child and for a parent is associated with a 0.654 (95% CI: 0.365 to 0.944) and 0.459 (95% CI: 0.147 to 0.771) higher GHQ-12 score than noncaregivers, respectively. Caring for a spouse is associated with the highest GHQ-12 score (β = 1.397, 95% CI: 1.187 to 1.608). Meanwhile, caregivers for “other relatives” and nonrelatives have similar depressive symptoms to noncaregivers.

#### Sensitivity analysis

A series of robustness checks were performed to ensure the reliability of the results. Given the fact that women are more likely to be unpaid caregivers and to seek support for their psychological well-being, one robustness check was conducted by restricting the sample to women (Mackenzie et al., 2016). The results are shown in Appendix Tables 4–7 in Appendix V. An alternative measure of subjective well-being was used to capture depression (when the GHQ-12 caseness score is larger than or equal to three). The random-effects logistic estimates for depression are shown in Appendix Tables 8 and 9.

The health status of care recipients may potentially influence both the hours of care provided and caregivers’ psychological well-being. The omission of this variable could cause endogeneity, biasing our fixed-effects estimates. Both BHPS and UKHLS are household-based surveys. Each adult member of the selected households was interviewed. This survey structure permits linking caregivers’ outcomes to the characteristics of the care recipients, including their self-reported health status. Robustness checks were performed specifically focusing on coresident caregivers. Those checks compared caregivers’ psychological well-being across various care relationships and by care intensity while controlling for the care recipient’s health status (refer to Appendix Tables 10–14). To manage missing data, we applied multiple imputation techniques and conducted analyses using these imputations (see Appendix Tables 15–18). Bootstrap standard errors were also calculated as part of robustness checks, with results shown in Appendix Tables 19–22. Last, to tackle potential issues arising from multiple comparisons, both the Bonferroni correction and Benjamini–Hochberg procedure were applied (see Appendix Tables 23–26). All these robustness checks yielded results that were consistent with the main findings described. Further details on these analyses can be found in Appendix V.

## Discussion and Conclusion

This study advances our understanding of the impact of informal caregiving on psychological well-being by using nationally representative longitudinal data over 27 years to identify the “tipping point” at which care intensity was negatively associated with caregivers’ psychological well-being. Previous work has overlooked the complexities of informal care, specifically the interplay between the location of care, care relationship, and care intensity and their influence on informal caregivers’ well-being. This study distinctively separated the influence of care relationship and location from that of care intensity.

Caring for more than 5 hours per week is associated with lower psychological well-being for both coresident and extraresident caregivers. Fewer hours of caregiving (under 5 hours per week) are only linked to higher psychological well-being for extraresident caregivers. This positive relationship may arise from improved closeness between caregiver and care recipient, or increased life satisfaction linked to doing what is considered dutiful or right (Litwin et al., 2014). A lack of geographical proximity could be accepted as a “legitimate excuse” to abstain from caring for someone living outside the household. Therefore, extraresident care involves more voluntary effort, improving caregivers’ psychological well-being (Marks et al., 2002). Conversely, caring for someone who lives within the household is more obligatory, and coresident caregivers are more likely to provide the types of support that involve more emotional or physical stress (Campbell & Martin-Matthews, 2000; De Koker, 2008). Living with a care recipient is also associated with a greater loss of social opportunities and distress (Cfas et al., 1998; Hirst, 2003).

When caregiver–care recipient relationship is accounted for, a negative relationship between psychological well-being and caring is found amongst those who care for primary kin (spouse, child, or parent), in line with Marks et al. (2002). Any level of care intensity is associated with lower psychological well-being for a caregiver of a spouse, and the thresholds are 50 hours per week for caregivers of a child and 5 hours per week for caregivers of a parent. However, caring is not associated with lower psychological well-being for caregivers of “other relatives” or nonrelatives, and light caring is linked to better well-being among them. As proposed by the hierarchical compensatory model of social support, caring for “other relatives” and nonrelatives is associated with a lower level of normative obligation compared to caring for primary kin (Robison et al., 2009). A higher degree of voluntary effort is linked to a greater level of personal satisfaction and purpose in life, improving caregivers’ psychological well-being.

Comparing the psychological well-being of extraresident and coresident caregivers, a significant difference is found only in the subsample who care for less than 10 hours per week. As care intensity increases, the positive well-being impact of care (which is found among extraresident caregivers) diminishes, offset by the physical and emotional stress associated with longer care hours (Brenna & Di Novi, 2016). There is no significant difference between the well-being of extraresident and coresident caregivers when care is provided for more than 10 hours per week. However, under all levels of care intensity, caregivers of “other relatives” or nonrelatives have higher levels of psychological well-being than those who care for primary kin. Consistent with Hong and Kim (2008) and Ott et al. (2007), we find that caring for a spouse is associated with the lowest level of psychological well-being. Partners and spouses sit at the top of the hierarchy of care obligations (Chappell et al., 2014; Lin et al., 2012; Penning & Wu, 2015). Embedded within the solemn promises of marriage vows, caring for a spouse stands as a fundamental commitment. Moreover, this duty often encompasses not only the inherent stresses of caregiving but also the consequential loss of financial and domestic labor support from the partner being cared for.

Some studies that fail to control for care intensity find that caring for a child is associated with lower psychological well-being (Penning & Wu, 2015). These results are unsurprising, given that caregivers of a child report the highest care intensity (see Figure 1). However, when we control for care hours, those who care for their child are found to have higher well-being than caregivers for a spouse or a parent. The inconsistent results in the literature could have been caused by a failure to better model the role of care intensity (closely linked to and varies across care relationships). This emphasizes the importance of considering caring intensity when examining the link between a caring relationship and the caregiver’s well-being.

### Limitations

There are limitations of the study that should be noted. Due to data limitations, we could not control for all the factors that may affect caregivers’ well-being. When those omitted factors are not time-invariant (such as preference for care arrangements), our fixed-effects estimates cannot be interpreted as causal. Our model fails to control for some important factors that might influence the normative hierarchies of care obligation, such as relationship quality and the support and resources that might be in place. Despite this, our study still provides a general picture of the caregivers’ psychological well-being by care location and care relationship.

The measure of care intensity is based on care time intervals, a limitation imposed by the data available. Therefore, it is not possible to pinpoint the precise threshold of care hours at which caregiving starts to be negatively associated with psychological well-being. Moreover, the aggregate measure of care hours does not account for the complexity and varying demands of different care tasks. This is a significant area for future research to build a more nuanced understanding of care intensity.

In addition, the study relies on the GHQ-12 score for the measurement of psychological well-being. Various other indicators of strain, stress, life satisfaction, or other aspects of psychological well-being might provide a more nuanced understanding of caregivers’ mental health. Furthermore, given the limitations in the available data, it is not possible to control for the duration of care, across which the well-being impact of care may vary. It is not possible to examine the heterogeneity among long-term caregivers and those who just started to provide care or have cared for a short period. Further research could analyze the short and longer-term impacts of caring on psychological well-being and how long the effects persist.

### Strengths

Despite these limitations, this study makes significant contributions to the literature as follows. The main aim of this study and the literature more broadly is to inform understanding of how the location of care, and caregiver–care recipient relationships, affect caregivers’ well-being, and to establish who is at greater risk of lower psychological well-being (Ennis et al., 2013). This study identifies thresholds of care intensity, which can be used to indicate the levels of care that are negatively associated with a caregiver’s well-being and the contexts in which positive interventions may be needed. The study has also compared the psychological well-being of caregivers who live with, and separately from, the person they care for, and among different caregiver–care recipient relationships, controlling for care intensity, aspects largely unexamined in existing studies (Andrén & Elmståhl, 2007; Chappell et al., 2014; Shahly et al., 2013).

The analyses support Hypotheses 1 and 2: the thresholds of care intensity at which care is negatively linked to caregivers’ well-being is lower among primary kin caregivers (e.g., caregivers of a spouse) and higher among those who look after other relationships (e.g., nonrelatives). Meanwhile, the primary kin caregivers have lower psychological well-being compared to caregivers of “other relatives” and nonrelatives. Extraresident caregivers report a higher level of psychological well-being than coresident caregivers when care is provided for less than 10 hours per week. At higher levels of care intensity, care location is not associated with caregivers’ psychological well-being anymore. The finding regarding the difference in caregivers’ well-being across care locations—as indicated within the extant literature—might be affected by care intensities (Cfas et al., 1998; Hirst, 2003).

### Recommendations and Implications

Future studies on the impacts of caring locations or caregiver–care recipient relationships (or other caring conditions more broadly) should consider the influence of care intensities and other structural factors that interact with the care settings. It is also important to investigate the mechanism behind the difference in caregiver’s psychological well-being across locations and relationships. For instance, if financial or household work strain mediates the well-being impact. This could be achieved with complimentary qualitative and ethnographic work. The norms and obligations of caregiving can be influenced by various factors, making them far from uniform across different groups. For instance, cultural background can play a substantial role in shaping expectations and attitudes toward caregiving. In some cultures, familial obligation toward providing care for older adults is deeply rooted, with adult children, particularly sons or daughters-in-law, often expected to take on the primary caregiver role (Zhang & Harper, 2022). Further research could explore these roles of religious and ethnic background and socioeconomic status.

Informal (unpaid) caregivers provide invaluable support to family members, friends, neighbors, and others in the absence of formal provision from health and social care services. They will continue to do so as societies age, service spending is cut, eligibility criteria for state-funded supports get stricter, and unmet need rises. Informal caregivers therefore often provide a lifeline to the people they care for and are integral to the health and social care (long-term care) sectors. The support they provide will necessarily be affected by their own psychological well-being. We demonstrate that the impact of care on psychological well-being is not experienced equally by people and is linked to the nature and circumstances of care, notably care location, relationships, and hours of care. Support for informal caregivers is crucial and providers of care and support services (local councils, trusts, and authorities) can use the findings of this study to understand who to target for urgent support. However, providers also need the resources to alleviate the pressures often experienced by caregivers, and until we see a radical reform of social care (long-term care), inequalities will persist.

## Supplementary Material

gbad166_suppl_Supplementary_Material
