# Supplementary material for: Insights Into Informal Caregivers’ Well-being: A Longitudinal Analysis of Care Intensity, Care Location, and Care Relationship
Source: J Gerontol B Psychol Sci Soc Sci. 2024 Jan 31;79(2):gbad166. doi: 10.1093/geronb/gbad166 (PMC10832593; doi:10.1093/geronb/gbad166)
Supplement: gbad166_suppl_Supplementary_Material [file gbad166_suppl_supplementary_material.docx]

**Appendix**

**Appendix I: BHPS and UKHLS**

The British Household Panel Survey (BHPS) and Understanding Society, the UK Household Longitudinal Study (UKHLS), are both household panel surveys, which follow the same individuals and households over time. The BHPS started in 1991 with a nationally representative sample of over 5,000 households, containing a total of 10,300 individuals from 250 areas of Great Britain. In 1999, an extra 1,500 households were added to the main sample from each of Scotland and Wales. Later, in 2001, an additional sample of 2,000 households from Northern Ireland was incorporated. As a result, from 2001 onwards, the panel became suitable for research covering the entire United Kingdom. The survey took place annually and covered a wide range of topics such as health, work, education, income, and social and political attitudes. Understanding Society, the UK Household Longitudinal Study, began in 2009 and expanded the BHPS. The UKHLS aimed to be representative of the population living in households in the UK. It started with a sample of approximately 40,000 households, making it the largest household panel survey of its kind.

Both surveys used a multi-stage stratified random sampling method. This involves first dividing the population into groups (or strata) and then randomly selecting samples from within each group. This method was chosen to ensure the sample is representative of the population across various dimensions (such as region, urban/rural location, and household composition). Panel attrition, or the ‘drop-out rate’, is a common issue in longitudinal studies, and the BHPS and UKHLS are no exceptions. This refers to the proportion of the sample that drops out of the study over time. This can be due to various reasons, such as respondents moving, losing interest, or passing away. The attrition rates for both the BHPS and UKHLS have not been constant over time, with some waves experiencing higher attrition rates than others. Detailed attrition rates for each wave are given in the technical reports for each survey, which can be accessed from the official websites of the BHPS and UKHLS (https://www.understandingsociety.ac.uk/).

**Appendix II: Definition of informal caregivers and caregiver-care recipient relationships**

**Definition of informal caregivers**

Respondents are defined as informal caregivers if they answer ‘yes’ to any of the following two questions:

“*Is there anyone living with you who is sick, disabled or elderly whom you look after or give special help to (for example, a sick, disabled or elderly relative, husband, wife or friend etc)?*”

or

“*Do you provide some regular service or help for any sick, disabled or elderly person not living with you?*”

**Care relationships**

1. **Provide care to someone outside the household**

Who is the person that you look after for if you cared for someone lives outside the household? (What is his/her relationship to you?)

1 parent/parent-in-law, 2 grandparent, 3 aunt/uncle, 4 other relatives, 5 friend or neighbour, 6 clients of voluntary organisation, 97 Other

1. **Provide care to someone live in the same household**

What is the relationship between you and the person that you look after for if you care for someone lives within the household?

1 husband/wife, 2 partner/cohabitee, 3 civil partner, 4 natural son/daughter, 5 adopted son/daughter, 6 foster child, 7 stepson/stepdaughter, 8 son-in-law/daughter-in-law, 9 natural parent, 10 adoptive parent, 11 foster parent, 12 step-parent, 13 parent-in-law, 14 natural brother/sister, 15 half-brother/sister, 16 step-brother/sister, 17 adopted brother/sister, 18 foster brother/sister, 19 brother/sister-in-law, 20 grand-child, 21 grand-parent, 22 cousin, 23 aunt/uncle, 24 niece/nephew, 25 other relative, 26 employee, 27 employer, 28 lodger/boarder/tenant, 29 landlord/landlady, 30 other non-relative

**Appendix III: Variable definitions**

Table A1: Variable Definitions

| **GHQ-12** | The Likert index for the General Health Questionnaire-12, ranging from 0-36. |
| --- | --- |
| **CareInt(0-4 hrs.)** | Dummy variable, equal to one if the respondent spends less than 5 hours per week on caring, and zero otherwise. |
| **CareInt(5-9 hrs.)** | Dummy variable, equal to one if the respondent spends 5-9 hours per week on caring, and zero otherwise. |
| **CareInt(10-19 hrs.)** | Dummy variable, equal to one if the respondent spends 10-19 hours per week on caring, and zero otherwise. |
| **CareInt(20-34 hrs.)** | Dummy variable, equal to one if the respondent spends 20-34 hours per week on caring, and zero otherwise. |
| **CareInt(35-49 hrs.)** | Dummy variable, equal to one if the respondent spends 35-49 hours per week on caring, and zero otherwise. |
| **CareInt(50+ hrs.)** | Dummy variable, equal to one if the respondent spends 50+ hours per week on caring, and zero otherwise. |
| **Degree** | Dummy variable, equal to one if the respondent has attained a degree, and zero otherwise. |
| **Age** | Age of the respondent. |
| **Married** | Dummy variable, equal to one if the respondent is married or cohabits with his/her partner, and zero otherwise. |
| **Widowed** | Dummy variable, equal to one if the respondent is widowed, and zero otherwise. |
| **Disable** | Dummy variable, equal to one if the respondent is disabled, and zero otherwise. |
| **Income** | The logarithm of the gross monthly personal income (imputed) at the 2016 price. |
| **Income square** | The square of the logarithm of the real gross monthly personal income. |
| **House Ownership** | Dummy variable, equal to 1 if the house where the respondent is living is owned outright, owned/being bought on a mortgage, or shared ownership, and zero otherwise. |
| **Household Size** | The number of people in the household. |
| **Work** | Dummy variable, equal to one if the respondent is self-employed or employed or an unpaid worker in a family business, and zero otherwise. |
| **Retired** | Dummy variable, equal to one if the respondent is retired, and zero otherwise |

**Appendix IV: Full results table**

**Table A2: Psychological wellbeing and caring location by caring intensity**

|  | **(1)** | **(2)** | **(3)** | **(4)** |
| --- | --- | --- | --- | --- |
|  | **< 10 hrs** | **[10 20)** | **[20 50)** | ≥ **50 hrs** |
|  | **ß (95% CI)** | **ß (95% CI)** | **ß (95% CI)** | **ß (95% CI)** |
|  |  |  |  |  |
| **Extra-residence** | -0.070* | 0.310*** | 0.552*** | 1.083*** |
|  | (-0.129 - -0.012) | (0.165 – 0.455) | (0.334 – 0.770) | (0.543 – 1.623) |
| **Co-residence** | 0.155* | 0.271** | 0.483*** | 1.023*** |
|  | (0.030 – 0.281) | (0.077 – 0.464) | (0.292 – 0.674) | (0.861 – 1.185) |
| **Degree** | 0.569*** | 0.560*** | 0.552*** | 0.570*** |
|  | (0.437 – 0.700) | (0.422 – 0.697) | (0.414 – 0.690) | (0.431 – 0.708) |
| **Age** | 0.018 | 0.004 | 0.017 | 0.006 |
|  | (-0.035 – 0.072) | (-0.052 – 0.061) | (-0.040 – 0.074) | (-0.051 – 0.063) |
| **Married** | 0.077 | 0.044 | 0.067 | 0.061 |
|  | (-0.001 – 0.154) | (-0.037 – 0.125) | (-0.014 – 0.148) | (-0.020 – 0.142) |
| **Widowed** | 1.380*** | 1.258*** | 1.352*** | 1.287*** |
|  | (1.102 – 1.658) | (0.962 – 1.554) | (1.058 – 1.647) | (1.001 – 1.573) |
| **Disabled** | 0.881*** | 0.883*** | 0.890*** | 0.887*** |
|  | (0.829 – 0.933) | (0.827 – 0.939) | (0.834 – 0.946) | (0.831 – 0.942) |
| **Income** | 0.068 | 0.056 | 0.058 | 0.059 |
|  | (-0.013 – 0.150) | (-0.030 – 0.143) | (-0.030 – 0.145) | (-0.028 – 0.146) |
| **Income square** | -0.004 | -0.003 | -0.003 | -0.003 |
|  | (-0.011 – 0.004) | (-0.011 – 0.005) | (-0.011 – 0.005) | (-0.011 – 0.005) |
| **House ownership** | -0.039 | -0.035 | -0.040 | -0.029 |
|  | (-0.116 – 0.038) | (-0.115 – 0.045) | (-0.120 – 0.040) | (-0.109 – 0.051) |
| **Household size** | 0.046*** | 0.041*** | 0.040** | 0.039** |
|  | (0.022 – 0.069) | (0.017 – 0.066) | (0.015 – 0.064) | (0.015 – 0.064) |
| **Work** | -0.878*** | -0.857*** | -0.866*** | -0.862*** |
|  | (-0.939 - -0.816) | (-0.921 - -0.792) | (-0.931 - -0.801) | (-0.927 - -0.798) |
| **Retired** | -1.437*** | -1.397*** | -1.412*** | -1.376*** |
|  | (-1.527 - -1.347) | (-1.494 - -1.301) | (-1.509 - -1.315) | (-1.472 - -1.280) |
| **Constant** | 9.654*** | 10.143*** | 9.770*** | 10.082*** |
|  | (8.120 – 11.188) | (8.543 – 11.744) | (8.165 – 11.375) | (8.469 – 11.695) |
|  |  |  |  |  |
| **Obs.** | 378,704 | 346,084 | 344,156 | 345,566 |
| **No. individuals** | 75,729 | 74,148 | 74,137 | 74,289 |

***Note***: A fixed-effects model is applied. 95% confidence interval is given in parentheses. Year dummies are included in all models, but their coefficients are not reported, for brevity.

****p*<0.001, ***p*<0.01, **p*<0.05

**Table A3: Psychological wellbeing and caregiver-care recipient relationship by care intensity**

|  | **(1)** | **(2)** | **(3)** | **(4)** |
| --- | --- | --- | --- | --- |
|  | **< 10 hrs** | **[10 20)** | **[20 50)** | ≥ **50 hrs** |
|  | **ß (95% CI)** | **ß (95% CI)** | **ß (95% CI)** | **ß (95% CI)** |
|  |  |  |  |  |
| **Spouse** | 0.321*** | 0.475*** | 0.781*** | 1.397*** |
|  | (0.161 – 0.481) | (0.224 – 0.725) | (0.531 – 1.030) | (1.187 – 1.608) |
| **Child** | 0.115 | -0.001 | 0.206 | 0.654*** |
|  | (-0.167 – 0.398) | (-0.399 – 0.398) | (-0.155 – 0.567) | (0.365 – 0.944) |
| **Parent** | 0.028 | 0.334*** | 0.531*** | 0.459** |
|  | (-0.044 – 0.100) | (0.183 – 0.485) | (0.323 – 0.740) | (0.147 – 0.771) |
| **Other relative** | -0.127* | 0.053 | 0.030 | -0.006 |
|  | (-0.237 - -0.017) | (-0.229 – 0.334) | (-0.328 – 0.387) | (-0.469 – 0.457) |
| **Non-relative** | -0.187*** | -0.395* | -0.558* | -0.565 |
|  | (-0.295 - -0.078) | (-0.752 - -0.037) | (-1.041 - -0.076) | (-1.171 – 0.040) |
| **Degree** | 0.569*** | 0.562*** | 0.551*** | 0.573*** |
|  | (0.437 – 0.700) | (0.424 – 0.699) | (0.413 – 0.689) | (0.435 – 0.712) |
| **Age** | 0.018 | 0.006 | 0.015 | 0.004 |
|  | (-0.035 – 0.072) | (-0.050 – 0.062) | (-0.041 – 0.072) | (-0.053 – 0.060) |
| **Married** | 0.073 | 0.043 | 0.067 | 0.051 |
|  | (-0.004 – 0.150) | (-0.038 – 0.124) | (-0.014 – 0.148) | (-0.030 – 0.132) |
| **Widowed** | 1.349*** | 1.250*** | 1.389*** | 1.288*** |
|  | (1.073 – 1.626) | (0.954 – 1.546) | (1.094 – 1.684) | (1.001 – 1.574) |
| **Disabled** | 0.881*** | 0.881*** | 0.892*** | 0.887*** |
|  | (0.829 – 0.933) | (0.825 – 0.937) | (0.836 – 0.948) | (0.832 – 0.943) |
| **Income** | 0.067 | 0.060 | 0.058 | 0.057 |
|  | (-0.015 – 0.148) | (-0.027 – 0.146) | (-0.029 – 0.145) | (-0.031 – 0.144) |
| **Income square** | -0.003 | -0.003 | -0.003 | -0.003 |
|  | (-0.011 – 0.004) | (-0.011 – 0.005) | (-0.011 – 0.005) | (-0.011 – 0.005) |
| **House ownership** | -0.042 | -0.035 | -0.039 | -0.028 |
|  | (-0.118 – 0.035) | (-0.115 – 0.044) | (-0.119 – 0.041) | (-0.108 – 0.052) |
| **Household size** | 0.046*** | 0.041** | 0.039** | 0.041*** |
|  | (0.023 – 0.069) | (0.016 – 0.065) | (0.014 – 0.063) | (0.017 – 0.066) |
| **Work** | -0.882*** | -0.855*** | -0.866*** | -0.861*** |
|  | (-0.944 - -0.821) | (-0.920 - -0.791) | (-0.931 - -0.801) | (-0.926 - -0.797) |
| **Retired** | -1.437*** | -1.392*** | -1.413*** | -1.384*** |
|  | (-1.526 - -1.347) | (-1.488 - -1.296) | (-1.510 - -1.317) | (-1.480 - -1.288) |
| **Constant** | 9.673*** | 10.092*** | 9.825*** | 10.157*** |
|  | (8.140 – 11.206) | (8.492 – 11.692) | (8.221 – 11.430) | (8.544 – 11.769) |
|  |  |  |  |  |
| **Obs.** | 380,403 | 346,712 | 344,826 | 346,268 |
| **No. individuals** | 75,967 | 74,297 | 74,288 | 74,429 |

***Note***: A fixed-effects model is applied. 95% confidence interval is given in parentheses. Year dummies are included in all models, but their coefficients are not reported, for brevity.

****p*<0.001, ***p*<0.01, **p*<0.05

**Appendix V: Sensitivity analysis**

**1.Robustness checks with subsamples of women and a new measurement of psychological wellbeing**

We conducted a series of robustness analyses with just a subsample of female caregivers (see Tables A4-A7), and with a different measurement of mental wellbeing, depression (see Tables A8-A9). All results are consistent with our main findings.

**2.Robustness check with care-recipient’s health status**

The health status of care recipients may potentially influence both the hours of care provided and caregivers' psychological wellbeing. The omission of this variable could cause endogeneity, biasing our fixed-effects estimates. In our initial analysis, we used non-caregivers as the reference group. This group, devoid of a care recipient, lacks any information regarding the health status of a care recipient, making it unfeasible to incorporate this aspect into the model.

Both the British Household Panel Survey (BHPS) and the UK Household Longitudinal Study (UKHLS) are household-based surveys. Each adult member of the selected households was interviewed. This survey structure permits linking caregivers' outcomes to the characteristics of the care recipients, including their self-reported health status. As a robustness check, we conducted a separate analysis that excluded non-caregivers and solely focused on co-resident caregivers, taking into account the health status of the care recipient. The health status variable is categorised into four segments: excellent, good, fair, and poor. For the analysis, we created four dummy variables, using "poor" health as the reference. The reference group in this analysis was those caring for non-relatives.

However, this approach significantly reduced the within-individual variance in care relationships by excluding non-caregivers from the sample. As a result, most of the fixed-effects coefficients for the care relationship were non-significant, as shown in Table A10. To address this, we carried out a random-effects analysis, the results of which are depicted in Table A11. The findings from the random-effects model align with our main conclusions, suggesting that primary kin caregiving is associated with lower levels of psychological wellbeing compared to caregiving in other relationships.

For an additional robustness check, we included non-caregivers as the reference group. In this case, we assigned a value of zero to the health status of the care recipient when the respondent was a non-caregiver. The results of this analysis, which are consistent with our primary findings, are presented in Tables A12 through A14.

**3.Robustness check with imputation**

To address the prevalence of missing data primarily found in the GHQ-12 (psychological well-being measure) and income variables, we utilized the Multiple Imputation by Chained Equations (MICE) technique, as facilitated by Stata 15 (White et al., 2021). This process involved implementing linear regression models that contained all variables from the initial model. We conducted this procedure over 20 imputation cycles. It's crucial to note that MICE operates under the assumption that the data are Missing at Random (MAR). In other words, the likelihood of a data point being missing relies solely on observed data and not on any unobserved data (Bhaskaran and Smeeth, 2014). This means that once the other variables in the dataset are controlled for, the missingness mechanism does not depend on any unobserved data. The diagnostic plots for the imputation model can be found in Figures A1 and A2. Other imputation diagnostic measures for each estimation, such as Relative Increases in Variance and Relative Efficiency can be provided upon request from the corresponding author. The results of the analysis using imputed data are detailed in Tables A15-A18. Upon comparison, these results align consistently with our original analysis.

**4.Robustness check with bootstrap standard error**

To enhance the robustness of our analysis and enable meaningful comparisons across groups, we employ bootstrap, a technique that estimates the variability of our statistic of interest, independent of sample size (Efron & Hastie, 2013). We apply bootstrap standard errors to our baseline models, ensuring the same sample size for each random draw across all groups. In this context, it is important to note that the sample size should not exceed the number of clusters, which in our case corresponds to the number of individuals. Thus, we set the sample size for random draws at 73,000. We performed 200 draws, using the seed value of 666 for replication of results. The results are reported in Tables A19-A22, in line with our main findings.

**5. Robustness check with Bonferroni correction and Benjamini-Hochberg procedure**

When conducting statistical analysis across multiple groups simultaneously, the likelihood of encountering a significant result purely by chance (known as a Type I error) increases substantially (Ranganathan, 2016). This phenomenon is often recognized as the "problem of multiple comparisons" or the "familywise error rate". To mitigate this concern, we used Bonferroni corrections (Bonferroni, 1936; VanderWeele & Mathur, 2019) and the Benjamini-Hochberg procedure (Haynes, 2013) in our analysis as a robustness check. The outcomes of these corrections are documented in Tables A23-A26. Notably, even after applying these rigorous adjustments for multiple comparisons, the key findings of our study remained consistent and robust.

**Table A4: Psychological wellbeing and caring intensity by location of care provision-female**

|  | **(1)** | **(2)** |
| --- | --- | --- |
|  | **Co-residence** | **Extra-residence** |
|  | **ß (95% CI)** | **ß (95% CI)** |
|  |  |  |
| **0-4 hrs.** | 0.272* | -0.125** |
|  | (0.039 - 0.505) | (-0.214 - -0.037) |
| **5-9 hrs.** | 0.287* | 0.169** |
|  | (0.027 - 0.547) | (0.054 - 0.284) |
| **10-19 hrs.** | 0.407** | 0.379*** |
|  | (0.153 - 0.661) | (0.228 - 0.530) |
| **20-34 hrs.** | 0.746*** | 0.594*** |
|  | (0.472 - 1.019) | (0.360 - 0.828) |
| **35-49 hrs.** | 0.654*** | 0.597** |
|  | (0.316 - 0.992) | (0.211 - 0.984) |
| **50+ hrs.** | 1.006*** | 0.955*** |
|  | (0.811 - 1.201) | (0.458 - 1.451) |
| **Degree** | 0.559*** | 0.493*** |
|  | (0.370 - 0.747) | (0.313 - 0.673) |
| **Age** | 0.045 | 0.044 |
|  | (-0.035 - 0.125) | (-0.031 - 0.119) |
| **Married** | -0.038 | -0.059 |
|  | (-0.144 - 0.069) | (-0.159 - 0.041) |
| **Widowed** | 1.253*** | 1.070*** |
|  | (0.914 - 1.593) | (0.774 - 1.366) |
| **Disabled** | 1.009*** | 0.955*** |
|  | (0.931 - 1.087) | (0.882 - 1.028) |
| **Income** | 0.079 | 0.134* |
|  | (-0.054 - 0.213) | (0.010 - 0.257) |
| **Income square** | -0.003 | -0.007 |
|  | (-0.015 - 0.009) | (-0.018 - 0.004) |
| **House Ownership** | -0.019 | -0.003 |
|  | (-0.132 - 0.093) | (-0.111 - 0.104) |
| **Household Size** | 0.010 | 0.020 |
|  | (-0.024 - 0.044) | (-0.012 - 0.053) |
| **Work** | -0.689*** | -0.723*** |
|  | (-0.771 - -0.607) | (-0.801 - -0.644) |
| **Retired** | -1.143*** | -1.236*** |
|  | (-1.273 - -1.013) | (-1.356 - -1.115) |
| **Constant** | 9.405*** | 9.230*** |
|  | (7.188 - 11.623) | (7.099 - 11.360) |
|  |  |  |
| **Obs.** | 193,153 | 213,421 |
| **No. individuals** | 40,262 | 41,749 |

***Note***: A fixed-effects model is applied. 95% confidence interval is given in parentheses. Year dummies are included in all models, but their coefficients are not reported, for brevity.

****p*<0.001, ***p*<0.01, **p*<0.05

**Table A5: Psychological wellbeing and caring intensity by caregiver-care recipient relationship-female**

|  | **(1)** | **(2)** | **(3)** | **(4)** | **(5)** |
| --- | --- | --- | --- | --- | --- |
|  | **Spouse** | **Child** | **Parent** | **Other relative** | **Non-relative** |
|  | **ß (95% CI)** | **ß (95% CI)** | **ß (95% CI)** | **ß (95% CI)** | **ß (95% CI)** |
|  |  |  |  |  |  |
| **0-4 hrs.** | 0.458** | 0.138 | -0.093 | -0.151 | -0.151 |
|  | (0.177 - 0.740) | (-0.342 - 0.619) | (-0.215 - 0.029) | (-0.327 - 0.026) | (-0.319 - 0.018) |
| **5-9 hrs.** | 0.570*** | -0.128 | 0.240*** | 0.041 | -0.179 |
|  | (0.243 - 0.897) | (-0.634 - 0.378) | (0.101 - 0.379) | (-0.205 - 0.287) | (-0.478 - 0.119) |
| **10-19 hrs.** | 0.732*** | 0.140 | 0.485*** | 0.099 | -0.347 |
|  | (0.402 - 1.061) | (-0.333 - 0.612) | (0.316 - 0.654) | (-0.227 - 0.425) | (-0.787 - 0.094) |
| **20-34 hrs.** | 1.171*** | 0.449 | 0.830*** | -0.033 | -0.197 |
|  | (0.801 - 1.541) | (-0.027 - 0.925) | (0.580 - 1.080) | (-0.512 - 0.447) | (-0.901 - 0.507) |
| **35-49 hrs.** | 1.212*** | 0.241 | 0.452* | 0.660 | -0.338 |
|  | (0.744 - 1.681) | (-0.286 - 0.767) | (0.058 - 0.846) | (-0.093 - 1.412) | (-1.485 - 0.810) |
| **50+ hrs.** | 1.434*** | 0.540*** | 0.967*** | 0.400 | -0.058 |
|  | (1.171 - 1.697) | (0.224 - 0.857) | (0.622 - 1.312) | (-0.205 - 1.006) | (-0.899 - 0.784) |
| **Degree** | 0.513*** | 0.515*** | 0.499*** | 0.512*** | 0.510*** |
|  | (0.323 - 0.704) | (0.324 - 0.706) | (0.314 - 0.683) | (0.326 - 0.699) | (0.321 - 0.698) |
| **Age** | 0.039 | 0.043 | 0.047 | 0.063 | 0.046 |
|  | (-0.042 - 0.120) | (-0.039 - 0.124) | (-0.030 - 0.125) | (-0.018 - 0.144) | (-0.035 - 0.127) |
| **Married** | -0.081 | -0.090 | -0.059 | -0.087 | -0.055 |
|  | (-0.190 - 0.027) | (-0.198 - 0.018) | (-0.164 - 0.046) | (-0.194 - 0.020) | (-0.163 - 0.052) |
| **Widowed** | 1.231*** | 1.204*** | 1.293*** | 1.128*** | 1.340*** |
|  | (0.875 - 1.588) | (0.837 - 1.570) | (0.939 - 1.647) | (0.761 - 1.495) | (0.972 - 1.708) |
| **Disable** | 0.994*** | 1.027*** | 0.982*** | 0.999*** | 0.996*** |
|  | (0.915 - 1.074) | (0.947 - 1.108) | (0.906 - 1.058) | (0.919 - 1.079) | (0.916 - 1.076) |
| **Income** | 0.073 | 0.076 | 0.116 | 0.099 | 0.061 |
|  | (-0.062 - 0.207) | (-0.059 - 0.212) | (-0.011 - 0.243) | (-0.033 - 0.232) | (-0.072 - 0.194) |
| **Income square** | -0.002 | -0.002 | -0.006 | -0.004 | -0.001 |
|  | (-0.014 - 0.010) | (-0.015 - 0.010) | (-0.017 - 0.006) | (-0.016 - 0.008) | (-0.013 - 0.011) |
| **House Ownership** | -0.008 | -0.023 | -0.017 | -0.019 | -0.025 |
|  | (-0.122 - 0.105) | (-0.137 - 0.091) | (-0.128 - 0.094) | (-0.132 - 0.093) | (-0.138 - 0.089) |
| **Household Size** | 0.021 | 0.015 | 0.011 | 0.013 | 0.031 |
|  | (-0.014 - 0.056) | (-0.020 - 0.050) | (-0.023 - 0.044) | (-0.022 - 0.047) | (-0.004 - 0.065) |
| **Work** | -0.669*** | -0.678*** | -0.710*** | -0.669*** | -0.676*** |
|  | (-0.753 - -0.586) | (-0.762 - -0.594) | (-0.790 - -0.629) | (-0.752 - -0.586) | (-0.759 - -0.593) |
| **Retired** | -1.113*** | -1.188*** | -1.252*** | -1.144*** | -1.150*** |
|  | (-1.244 - -0.982) | (-1.325 - -1.052) | (-1.379 - -1.125) | (-1.278 - -1.010) | (-1.283 - -1.017) |
| **Constant** | 9.545*** | 9.508*** | 9.241*** | 8.877*** | 9.343*** |
|  | (7.294 - 11.797) | (7.277 - 11.740) | (7.094 - 11.388) | (6.676 - 11.078) | (7.106 - 11.579) |
|  |  |  |  |  |  |
| **Obs.** | 187,321 | 184,759 | 201,181 | 187,980 | 186,414 |
| **No. individuals** | 39,419 | 39,073 | 40,154 | 39,418 | 39,037 |

***Note***: A fixed-effects model is applied. 95% confidence interval is given in parentheses. Year dummies are included in all models, but their coefficients are not reported, for brevity.

****p*<0.001, ***p*<0.01, **p*<0.05

**Table A6: Psychological wellbeing and caring location by caring intensity-female**

|  | **(1)** | **(2)** | **(3)** | **(4)** |
| --- | --- | --- | --- | --- |
|  | **< 10 hrs** | **[10 20)** | **[20 50)** | ≥ **50 hrs** |
|  | **ß (95% CI)** | **ß (95% CI)** | **ß (95% CI)** | **ß (95% CI)** |
|  |  |  |  |  |
| **Extra-residence** | -0.034 | 0.460*** | 0.616*** | 1.255*** |
|  | (-0.115 - 0.046) | (0.279 - 0.640) | (0.354 - 0.879) | (0.602 - 1.909) |
| **Co-residence** | 0.301** | 0.081 | 0.577*** | 1.068*** |
|  | (0.111 - 0.491) | (-0.208 - 0.370) | (0.312 - 0.842) | (0.852 - 1.283) |
| **Degree** | 0.515*** | 0.490*** | 0.487*** | 0.517*** |
|  | (0.334 - 0.695) | (0.301 - 0.680) | (0.296 - 0.678) | (0.326 - 0.708) |
| **Age** | 0.050 | 0.044 | 0.053 | 0.041 |
|  | (-0.026 - 0.127) | (-0.038 - 0.125) | (-0.029 - 0.134) | (-0.041 - 0.122) |
| **Married** | -0.044 | -0.091 | -0.063 | -0.069 |
|  | (-0.147 - 0.060) | (-0.199 - 0.017) | (-0.172 - 0.045) | (-0.177 - 0.039) |
| **Widowed** | 1.309*** | 1.156*** | 1.310*** | 1.240*** |
|  | (0.963 - 1.655) | (0.787 - 1.526) | (0.943 - 1.676) | (0.884 - 1.596) |
| **Disabled** | 0.977*** | 0.998*** | 1.007*** | 0.999*** |
|  | (0.902 - 1.052) | (0.918 - 1.079) | (0.926 - 1.088) | (0.918 - 1.079) |
| **Income** | 0.099 | 0.082 | 0.080 | 0.087 |
|  | (-0.027 - 0.224) | (-0.051 - 0.216) | (-0.055 - 0.216) | (-0.048 - 0.222) |
| **Income square** | -0.004 | -0.003 | -0.003 | -0.004 |
|  | (-0.015 - 0.007) | (-0.015 - 0.009) | (-0.015 - 0.009) | (-0.016 - 0.008) |
| **House ownership** | -0.032 | -0.018 | -0.025 | -0.008 |
|  | (-0.141 - 0.077) | (-0.132 - 0.096) | (-0.140 - 0.089) | (-0.122 - 0.106) |
| **Household size** | 0.021 | 0.020 | 0.014 | 0.013 |
|  | (-0.012 - 0.055) | (-0.015 - 0.055) | (-0.021 - 0.049) | (-0.022 - 0.049) |
| **Work** | -0.700*** | -0.666*** | -0.679*** | -0.680*** |
|  | (-0.779 - -0.620) | (-0.749 - -0.582) | (-0.763 - -0.595) | (-0.764 - -0.596) |
| **Retired** | -1.197*** | -1.164*** | -1.184*** | -1.134*** |
|  | (-1.322 - -1.072) | (-1.298 - -1.030) | (-1.320 - -1.049) | (-1.268 - -0.999) |
| **Constant** | 9.113*** | 9.446*** | 9.181*** | 9.496*** |
|  | (6.979 - 11.246) | (7.219 - 11.674) | (6.943 - 11.419) | (7.249 - 11.743) |
|  |  |  |  |  |
| **Obs.** | 204,612 | 186,087 | 184,805 | 185,733 |
| **No. individuals** | 40,295 | 39,456 | 39,433 | 39,549 |

***Note***: A fixed-effects model is applied. 95% confidence interval is given in parentheses. Year dummies are included in all models, but their coefficients are not reported, for brevity.

****p*<0.001, ***p*<0.01, **p*<0.05

**Table A7: Psychological wellbeing and caregiver-care recipient relationship by care intensity-female**

|  | **(1)** | **(2)** | **(3)** | **(4)** |
| --- | --- | --- | --- | --- |
|  | **< 10 hrs** | **[10 20)** | **[20 50)** | **>= 50 hrs** |
|  | **ß (95% CI)** | **ß (95% CI)** | **ß (95% CI)** | **ß (95% CI)** |
|  |  |  |  |  |
| **Spouse** | 0.448*** | 0.518** | 1.019*** | 1.559*** |
|  | (0.212 - 0.684) | (0.136 - 0.901) | (0.652 - 1.386) | (1.265 - 1.853) |
| **Child** | 0.119 | -0.217 | 0.351 | 0.534** |
|  | (-0.273 - 0.511) | (-0.766 - 0.331) | (-0.102 - 0.805) | (0.183 - 0.884) |
| **Parent** | 0.051 | 0.434*** | 0.686*** | 0.854*** |
|  | (-0.050 - 0.152) | (0.243 - 0.624) | (0.429 - 0.943) | (0.461 - 1.247) |
| **Other relative** | -0.111 | 0.067 | -0.015 | 0.181 |
|  | (-0.261 - 0.038) | (-0.292 - 0.426) | (-0.472 - 0.441) | (-0.407 - 0.770) |
| **Non-relative** | -0.165* | -0.500* | -0.637 | -0.416 |
|  | (-0.315 - -0.015) | (-0.970 - -0.029) | (-1.291 - 0.017) | (-1.226 - 0.394) |
| **Degree** | 0.519*** | 0.498*** | 0.488*** | 0.521*** |
|  | (0.339 - 0.699) | (0.308 - 0.687) | (0.298 - 0.679) | (0.330 - 0.712) |
| **Age** | 0.051 | 0.046 | 0.051 | 0.040 |
|  | (-0.026 - 0.127) | (-0.035 - 0.128) | (-0.030 - 0.133) | (-0.041 - 0.122) |
| **Married** | -0.052 | -0.092 | -0.065 | -0.080 |
|  | (-0.155 - 0.051) | (-0.200 - 0.015) | (-0.173 - 0.044) | (-0.188 - 0.028) |
| **Widowed** | 1.284*** | 1.154*** | 1.355*** | 1.235*** |
|  | (0.939 - 1.629) | (0.785 - 1.523) | (0.989 - 1.722) | (0.878 - 1.592) |
| **Disabled** | 0.976*** | 0.995*** | 1.009*** | 1.000*** |
|  | (0.901 - 1.051) | (0.915 - 1.076) | (0.929 - 1.090) | (0.920 - 1.080) |
| **Income** | 0.094 | 0.091 | 0.083 | 0.083 |
|  | (-0.031 - 0.220) | (-0.043 - 0.225) | (-0.052 - 0.218) | (-0.052 - 0.219) |
| **Income square** | -0.004 | -0.004 | -0.003 | -0.003 |
|  | (-0.015 - 0.008) | (-0.016 - 0.008) | (-0.015 - 0.009) | (-0.015 - 0.009) |
| **House ownership** | -0.034 | -0.014 | -0.024 | -0.007 |
|  | (-0.143 - 0.075) | (-0.128 - 0.100) | (-0.138 - 0.090) | (-0.121 - 0.107) |
| **Household size** | 0.021 | 0.018 | 0.012 | 0.016 |
|  | (-0.012 - 0.054) | (-0.017 - 0.053) | (-0.023 - 0.047) | (-0.019 - 0.051) |
| **Work** | -0.706*** | -0.665*** | -0.679*** | -0.679*** |
|  | (-0.785 - -0.626) | (-0.749 - -0.582) | (-0.763 - -0.595) | (-0.763 - -0.595) |
| **Retired** | -1.201*** | -1.157*** | -1.193*** | -1.146*** |
|  | (-1.325 - -1.076) | (-1.291 - -1.023) | (-1.328 - -1.058) | (-1.280 - -1.012) |
| **Constant** | 9.142*** | 9.356*** | 9.224*** | 9.541*** |
|  | (7.012 - 11.273) | (7.129 - 11.582) | (6.987 - 11.462) | (7.295 - 11.787) |
|  |  |  |  |  |
| **Obs.** | 205,593 | 186,498 | 185,213 | 186,235 |
| **No. individuals** | 40,426 | 39,560 | 39,529 | 39,646 |

***Note***: A fixed-effects model is applied. 95% confidence interval is given in parentheses. Year dummies are included in all models, but their coefficients are not reported, for brevity.

****p*<0.001, ***p*<0.01, **p*<0.05

**Table A8: Depression and caring location by care intensity**

|  | **(1)** | **(2)** | **(3)** | **(4)** |
| --- | --- | --- | --- | --- |
|  | **< 10 hrs**  **OR**  **(95% CI)** | **[10 20)**  **OR**  **(95% CI)** | **[20 50)**  **OR**  **(95% CI)** | ≥ **50 hrs**  **OR**  **(95% CI)** |
|  |  |  |  |  |
| **Extra-residence** | 0.925*** | 1.238*** | 1.450*** | 1.464** |
|  | (0.894 - 0.957) | (1.147 - 1.336) | (1.301 - 1.617) | (1.121 - 1.913) |
| **Co-residence** | 1.091** | 1.270*** | 1.508*** | 1.806*** |
|  | (1.022 - 1.165) | (1.158 - 1.392) | (1.387 - 1.640) | (1.688 - 1.933) |
| **Degree** | 1.075*** | 1.069*** | 1.075*** | 1.072*** |
|  | (1.038 - 1.113) | (1.032 - 1.108) | (1.037 - 1.114) | (1.035 - 1.111) |
| **Age** | 1.001* | 1.001* | 1.001* | 1.001** |
|  | (1.000 - 1.002) | (1.000 - 1.002) | (1.000 - 1.002) | (1.000 - 1.003) |
| **Married** | 0.912*** | 0.906*** | 0.907*** | 0.907*** |
|  | (0.881 - 0.945) | (0.874 - 0.939) | (0.875 - 0.941) | (0.875 - 0.941) |
| **Widowed** | 1.988*** | 1.968*** | 1.944*** | 1.902*** |
|  | (1.806 - 2.189) | (1.784 - 2.171) | (1.763 - 2.145) | (1.726 - 2.096) |
| **Disabled** | 1.951*** | 2.001*** | 2.004*** | 1.983*** |
|  | (1.900 - 2.004) | (1.946 - 2.057) | (1.949 - 2.060) | (1.930 - 2.039) |
| **Income** | 1.193*** | 1.191*** | 1.188*** | 1.182*** |
|  | (1.143 - 1.246) | (1.139 - 1.246) | (1.136 - 1.242) | (1.130 - 1.236) |
| **Income square** | 0.983*** | 0.984*** | 0.984*** | 0.984*** |
|  | (0.980 - 0.987) | (0.980 - 0.987) | (0.980 - 0.987) | (0.980 - 0.988) |
| **House ownership** | 0.676*** | 0.674*** | 0.675*** | 0.675*** |
|  | (0.657 - 0.695) | (0.656 - 0.694) | (0.656 - 0.695) | (0.656 - 0.694) |
| **Household size** | 1.052*** | 1.052*** | 1.051*** | 1.050*** |
|  | (1.042 - 1.062) | (1.042 - 1.062) | (1.041 - 1.061) | (1.040 - 1.060) |
| **Work** | 0.586*** | 0.584*** | 0.583*** | 0.585*** |
|  | (0.568 - 0.604) | (0.566 - 0.603) | (0.565 - 0.601) | (0.567 - 0.603) |
| **Retired** | 0.496*** | 0.502*** | 0.505*** | 0.513*** |
|  | (0.474 - 0.520) | (0.479 - 0.527) | (0.481 - 0.530) | (0.489 - 0.538) |
| **Constant** | 0.272*** | 0.278*** | 0.283*** | 0.288*** |
|  | (0.233 - 0.317) | (0.237 - 0.326) | (0.241 - 0.332) | (0.246 - 0.338) |
|  |  |  |  |  |
| **Obs.** | 405,224 | 371,125 | 369,175 | 370,912 |
| **No. individuals** | 79,296 | 77,733 | 77,738 | 77,933 |

***Note***: We use a random-effects logistic model, and the odds ratios are reported. 95% confidence interval is given in parentheses. Year dummies are included in all models, but their coefficients are not reported, for brevity.

****p*<0.001, ***p*<0.01, **p*<0.05

**Table A9: Depression and caregiver-care recipient relationship by care intensity**

|  | **(1)** | **(2)** | **(3)** | **(4)** |
| --- | --- | --- | --- | --- |
|  | **< 10 hrs**  **OR**  **(95% CI)** | **[10 20)**  **OR**  **(95% CI)** | **[20 50)**  **OR**  **(95% CI)** | **>= 50 hrs**  **OR**  **(95% CI)** |
|  |  |  |  |  |
| **Spouse** | 1.205*** | 1.510*** | 1.597*** | 2.019*** |
|  | (1.106 - 1.314) | (1.338 - 1.703) | (1.430 - 1.785) | (1.845 - 2.210) |
| **Child** | 1.071 | 1.119 | 1.214* | 1.640*** |
|  | (0.929 - 1.234) | (0.927 - 1.350) | (1.040 - 1.416) | (1.459 - 1.844) |
| **Parent** | 0.985 | 1.213*** | 1.393*** | 1.228** |
|  | (0.945 - 1.026) | (1.124 - 1.310) | (1.261 - 1.539) | (1.069 - 1.411) |
| **Other relative** | 0.952 | 1.071 | 1.175 | 1.184 |
|  | (0.895 - 1.013) | (0.929 - 1.235) | (0.993 - 1.391) | (0.958 - 1.464) |
| **Non-relative** | 0.854*** | 0.991 | 0.942 | 0.775 |
|  | (0.799 - 0.912) | (0.814 - 1.207) | (0.739 - 1.201) | (0.575 - 1.044) |
| **Degree** | 1.072*** | 1.068*** | 1.074*** | 1.072*** |
|  | (1.036 - 1.110) | (1.031 - 1.107) | (1.036 - 1.113) | (1.035 - 1.111) |
| **Age** | 1.001 | 1.001* | 1.001* | 1.001** |
|  | (1.000 - 1.002) | (1.000 - 1.002) | (1.000 - 1.002) | (1.000 - 1.003) |
| **Married** | 0.911*** | 0.904*** | 0.907*** | 0.902*** |
|  | (0.880 - 0.944) | (0.871 - 0.937) | (0.875 - 0.941) | (0.870 - 0.935) |
| **Widowed** | 1.993*** | 1.970*** | 1.958*** | 1.902*** |
|  | (1.810 - 2.193) | (1.785 - 2.173) | (1.775 - 2.159) | (1.725 - 2.096) |
| **Disabled** | 1.948*** | 1.998*** | 2.004*** | 1.984*** |
|  | (1.897 - 2.000) | (1.944 - 2.054) | (1.949 - 2.060) | (1.930 - 2.039) |
| **Income** | 1.193*** | 1.191*** | 1.188*** | 1.183*** |
|  | (1.143 - 1.245) | (1.139 - 1.246) | (1.136 - 1.242) | (1.131 - 1.237) |
| **Income square** | 0.983*** | 0.984*** | 0.984*** | 0.984*** |
|  | (0.980 - 0.987) | (0.980 - 0.987) | (0.980 - 0.987) | (0.980 - 0.988) |
| **House ownership** | 0.676*** | 0.675*** | 0.675*** | 0.675*** |
|  | (0.658 - 0.695) | (0.656 - 0.695) | (0.656 - 0.695) | (0.656 - 0.694) |
| **Household size** | 1.053*** | 1.052*** | 1.052*** | 1.051*** |
|  | (1.043 - 1.063) | (1.042 - 1.062) | (1.042 - 1.062) | (1.041 - 1.061) |
| **Work** | 0.584*** | 0.584*** | 0.582*** | 0.584*** |
|  | (0.567 - 0.602) | (0.566 - 0.603) | (0.564 - 0.601) | (0.566 - 0.603) |
| **Retired** | 0.498*** | 0.501*** | 0.503*** | 0.509*** |
|  | (0.475 - 0.521) | (0.478 - 0.526) | (0.480 - 0.528) | (0.485 - 0.534) |
| **Constant** | 0.274*** | 0.279*** | 0.283*** | 0.287*** |
|  | (0.235 - 0.319) | (0.238 - 0.327) | (0.241 - 0.331) | (0.245 - 0.337) |
|  |  |  |  |  |
| **Obs.** | 407,018 | 371,796 | 369,905 | 371,660 |
| **No. individuals** | 79,532 | 77,883 | 77,891 | 78,067 |

***Note***: We use a random-effects logistic model, and the odds ratios are reported. 95% confidence interval is given in parentheses. Year dummies are included in all models, but their coefficients are not reported, for brevity.

****p*<0.001, ***p*<0.01, **p*<0.05

**Table A10: Psychological wellbeing and caregiver-care recipient relationship by care intensity, with care-recipient’s health status, FE**

|  | **(1)** | **(2)** | **(3)** | **(4)** | **(1)** |
| --- | --- | --- | --- | --- | --- |
|  | All | **< 10 hrs** | **[10 20)** | **[20 50)** | **≥ 50 hrs** |
|  | **ß (95% CI)** | **ß (95% CI)** | **ß (95% CI)** | **ß (95% CI)** | **ß (95% CI)** |
|  |  |  |  |  |  |
| **Spouse** | 0.290 | -0.589 | 5.560* | 0.975 | 1.410 |
|  | (-0.762 - 1.341) | (-2.759 - 1.581) | (1.000 - 10.120) | (-2.919 - 4.868) | (-2.075 - 4.895) |
| **Child** | 0.245 | 0.814 | 6.257 | 0.391 | -2.239 |
|  | (-1.309 - 1.798) | (-2.662 - 4.289) | (-0.161 - 12.676) | (-4.843 - 5.625) | (-6.626 - 2.148) |
| **Parent** | -0.223 | 0.668 | -1.564 | 1.954* | -0.415 |
|  | (-0.645 - 0.199) | (-0.154 - 1.489) | (-3.271 - 0.143) | (0.263 - 3.646) | (-1.580 - 0.750) |
| **Other relative** | -0.013 | 0.427 | 0.139 | 0.379 | -0.946 |
|  | (-0.428 - 0.402) | (-0.384 - 1.238) | (-2.066 - 2.344) | (-1.264 - 2.021) | (-2.042 - 0.150) |
| **Excellent**  **(care-recipient)** | -0.388 | -0.391 | -1.965 | -1.107 | -1.436* |
|  | (-1.024 - 0.247) | (-1.668 - 0.885) | (-5.583 - 1.654) | (-3.496 - 1.282) | (-2.860 - -0.013) |
| **Good**  **(care-recipient)** | -0.295** | -0.240 | -0.323 | -0.385 | -0.438 |
|  | (-0.518 - -0.072) | (-0.698 - 0.219) | (-1.382 - 0.736) | (-1.213 - 0.442) | (-0.944 - 0.067) |
| **Fair**  **(care-recipient)** | -0.302** | -0.221 | 0.194 | -0.439 | -0.265 |
|  | (-0.489 - -0.115) | (-0.619 - 0.177) | (-0.669 - 1.057) | (-1.146 - 0.268) | (-0.691 - 0.161) |
| **Degree** | 1.259 | -0.404 | 0.313 | 1.587 |  |
|  | (-0.294 - 2.812) | (-2.894 - 2.085) | (-7.272 - 7.898) | (-6.843 - 10.016) |  |
| **Age** | -0.052 | -0.126 | -1.032 | 0.999 | -0.094 |
|  | (-0.363 - 0.260) | (-0.789 - 0.536) | (-2.473 - 0.409) | (-0.062 - 2.060) | (-0.817 - 0.630) |
| **Married** | 0.236 | 1.735* | -1.355 | 1.215 | -0.559 |
|  | (-0.490 - 0.963) | (0.379 - 3.091) | (-4.468 - 1.758) | (-2.003 - 4.433) | (-2.607 - 1.489) |
| **Widowed** | 1.517* | 2.653 | -3.045 | 7.379** | 3.094* |
|  | (0.142 - 2.893) | (-0.781 - 6.087) | (-10.030 - 3.939) | (1.862 - 12.897) | (0.381 - 5.807) |
| **Disabled** | 1.158*** | 1.368*** | 0.818 | 1.377** | 0.948** |
|  | (0.901 - 1.415) | (0.816 - 1.919) | (-0.405 - 2.041) | (0.407 - 2.347) | (0.349 - 1.546) |
| **Income** | 0.089 | -0.495 | 0.456 | -1.090 | 2.292** |
|  | (-0.484 - 0.663) | (-1.621 - 0.630) | (-2.352 - 3.265) | (-3.350 - 1.169) | (0.753 - 3.831) |
| **Income square** | -0.022 | 0.035 | -0.002 | 0.055 | -0.224** |
|  | (-0.073 - 0.028) | (-0.065 - 0.135) | (-0.246 - 0.243) | (-0.139 - 0.249) | (-0.357 - -0.091) |
| **House Ownership** | 0.067 | -0.258 | -0.121 | -0.569 | 0.717 |
|  | (-0.507 - 0.641) | (-1.553 - 1.036) | (-2.735 - 2.493) | (-2.501 - 1.363) | (-0.656 - 2.090) |
| **Household Size** | 0.019 | -0.062 | 0.215 | 0.081 | -0.071 |
|  | (-0.159 - 0.198) | (-0.408 - 0.283) | (-0.759 - 1.190) | (-0.480 - 0.642) | (-0.518 - 0.376) |
| **Work** | -0.865*** | -1.643*** | -1.164 | -0.533 | -0.231 |
|  | (-1.244 - -0.486) | (-2.417 - -0.870) | (-2.879 - 0.552) | (-2.018 - 0.951) | (-1.118 - 0.656) |
| **Retired** | -0.863*** | -1.974*** | -1.007 | -0.759 | -0.170 |
|  | (-1.231 - -0.495) | (-2.878 - -1.070) | (-2.809 - 0.795) | (-2.164 - 0.646) | (-0.896 - 0.556) |
| **Constant** | 13.901* | 16.080 | 50.127 | -24.560 | 11.901 |
|  | (1.289 - 26.512) | (-7.788 - 39.949) | (-5.606 - 105.861) | (-67.888 - 18.769) | (-21.455 - 45.258) |
|  |  |  |  |  |  |
| **Obs.** | 17,934 | 5,709 | 2,347 | 2,706 | 4,167 |
| **No. individuals** | 7,188 | 3,523 | 1,772 | 1,825 | 2,136 |

***Note***: A fixed-effects model is applied. 95% confidence interval is given in parentheses. Year dummies are included in all models, but their coefficients are not reported, for brevity.

****p*<0.001, ***p*<0.01, **p*<0.05

**Table A11: Psychological wellbeing and caregiver-care recipient relationship by care intensity, with care-recipient’s health status, RE**

|  | **(1)** | **(2)** | **(3)** | **(4)** | **(5)** |
| --- | --- | --- | --- | --- | --- |
|  | **All** | **< 10 hrs** | **[10 20)** | **[20 50)** | **≥ 50 hrs** |
|  | **ß (95% CI)** | **ß (95% CI)** | **ß (95% CI)** | **ß (95% CI)** | **ß (95% CI)** |
|  |  |  |  |  |  |
| **Spouse** | 1.003*** | 0.644* | 1.133* | 1.329* | 1.350** |
|  | (0.589 - 1.417) | (0.054 - 1.234) | (0.177 - 2.089) | (0.294 - 2.364) | (0.348 - 2.353) |
| **Child** | 1.843*** | 1.463*** | 1.937** | 2.090*** | 2.619*** |
|  | (1.314 - 2.372) | (0.688 - 2.238) | (0.686 - 3.188) | (0.878 - 3.303) | (1.413 - 3.825) |
| **Parent** | 0.082 | 0.478* | 0.043 | 0.880* | 0.242 |
|  | (-0.223 - 0.386) | (0.029 - 0.927) | (-0.715 - 0.801) | (0.070 - 1.689) | (-0.552 - 1.036) |
| **Other relative** | 0.187 | 0.808** | 0.462 | 0.114 | 0.051 |
|  | (-0.136 - 0.510) | (0.316 - 1.301) | (-0.395 - 1.320) | (-0.772 - 1.000) | (-0.753 - 0.856) |
| **Excellent**  **(care-recipient)** | -1.022*** | -1.425** | -2.191* | -0.640 | -1.629** |
|  | (-1.552 - -0.492) | (-2.276 - -0.573) | (-3.926 - -0.456) | (-2.212 - 0.933) | (-2.819 - -0.438) |
| **Good**  **(care-recipient)** | -0.504*** | -0.620*** | -0.647* | -0.339 | -0.549** |
|  | (-0.687 - -0.321) | (-0.928 - -0.312) | (-1.181 - -0.113) | (-0.868 - 0.189) | (-0.945 - -0.152) |
| **Fair**  **(care-recipient)** | -0.400*** | -0.424** | -0.083 | -0.466* | -0.364* |
|  | (-0.561 - -0.239) | (-0.707 - -0.141) | (-0.565 - 0.399) | (-0.931 - -0.001) | (-0.709 - -0.019) |
| **Degree** | -0.181 | -0.260 | -0.100 | -0.287 | 0.682 |
|  | (-0.584 - 0.222) | (-0.770 - 0.249) | (-0.966 - 0.766) | (-1.326 - 0.753) | (-0.343 - 1.708) |
| **Age** | -0.014** | -0.001 | -0.003 | -0.022 | -0.057*** |
|  | (-0.024 - -0.004) | (-0.016 - 0.014) | (-0.028 - 0.022) | (-0.048 - 0.004) | (-0.079 - -0.034) |
| **Married** | 0.394 | 1.039*** | 0.181 | 0.626 | -0.074 |
|  | (-0.006 - 0.794) | (0.462 - 1.617) | (-0.758 - 1.120) | (-0.395 - 1.648) | (-1.038 - 0.890) |
| **Widowed** | 0.748 | 1.095 | -1.168 | 0.818 | 1.099 |
|  | (-0.082 - 1.578) | (-0.303 - 2.493) | (-3.546 - 1.209) | (-1.164 - 2.800) | (-0.511 - 2.708) |
| **Disabled** | 2.020*** | 2.105*** | 2.765*** | 2.800*** | 1.962*** |
|  | (1.821 - 2.218) | (1.771 - 2.439) | (2.211 - 3.319) | (2.270 - 3.331) | (1.539 - 2.385) |
| **Income** | 0.447 | 0.447 | 0.618 | -0.190 | 1.128 |
|  | (-0.001 - 0.894) | (-0.236 - 1.129) | (-0.545 - 1.781) | (-1.520 - 1.139) | (-0.044 - 2.300) |
| **Income square** | -0.046* | -0.047 | -0.057 | -0.011 | -0.106* |
|  | (-0.085 - -0.008) | (-0.105 - 0.012) | (-0.157 - 0.043) | (-0.123 - 0.100) | (-0.205 - -0.007) |
| **House Ownership** | -0.345** | -0.217 | -0.014 | -0.195 | -0.222 |
|  | (-0.594 - -0.097) | (-0.594 - 0.160) | (-0.589 - 0.562) | (-0.762 - 0.372) | (-0.728 - 0.284) |
| **Household Size** | -0.056 | -0.078 | -0.005 | -0.049 | -0.096 |
|  | (-0.151 - 0.038) | (-0.216 - 0.059) | (-0.228 - 0.217) | (-0.277 - 0.179) | (-0.316 - 0.124) |
| **Work** | -1.488*** | -1.599*** | -1.806*** | -1.587*** | -0.797* |
|  | (-1.763 - -1.214) | (-2.047 - -1.151) | (-2.549 - -1.062) | (-2.311 - -0.863) | (-1.415 - -0.179) |
| **Retired** | -1.608*** | -2.144*** | -3.065*** | -1.700*** | -0.800** |
|  | (-1.898 - -1.317) | (-2.682 - -1.605) | (-3.918 - -2.212) | (-2.490 - -0.909) | (-1.358 - -0.241) |
| **Constant** | 12.187*** | 10.346*** | 12.094*** | 14.403*** | 13.369*** |
|  | (10.592 - 13.783) | (7.826 - 12.865) | (7.793 - 16.395) | (9.520 - 19.286) | (9.271 - 17.467) |
|  |  |  |  |  |  |
| **Obs.** | 17,934 | 5,709 | 2,347 | 2,706 | 4,167 |
| **No. individuals** | 7,188 | 3,523 | 1,772 | 1,825 | 2,136 |

***Note***: A random-effects model is applied. 95% confidence interval is given in parentheses. Year dummies are included in all models, but their coefficients are not reported, for brevity.

****p*<0.001, ***p*<0.01, **p*<0.05

**Table A12: Psychological wellbeing and caring intensity for co-resident carers, with care-recipient’s health status**

|  | **Co-residence** |
| --- | --- |
|  | **ß (95% CI)** |
|  |  |
| **0-4 hrs.** | 0.393*** |
|  | (0.164 - 0.622) |
| **5-9 hrs.** | 0.487*** |
|  | (0.239 - 0.736) |
| **10-19 hrs.** | 0.635*** |
|  | (0.384 - 0.885) |
| **20-34 hrs.** | 0.880*** |
|  | (0.603 - 1.156) |
| **35-49 hrs.** | 0.835*** |
|  | (0.475 - 1.195) |
| **50+ hrs.** | 1.125*** |
|  | (0.900 - 1.350) |
| **Excellent (care-recipient)** | -0.826** |
|  | (-1.420 - -0.231) |
| **Good (care-recipient)** | -0.249* |
|  | (-0.442 - -0.056) |
| **Fair (care-recipient)** | -0.284** |
|  | (-0.459 - -0.108) |
| **Degree** | 0.581*** |
|  | (0.444 - 0.719) |
| **Age** | 0.006 |
|  | (-0.050 - 0.062) |
| **Married** | 0.079 |
|  | (-0.001 - 0.160) |
| **Widowed** | 1.331*** |
|  | (1.046 - 1.616) |
| **Disabled** | 0.894*** |
|  | (0.839 - 0.949) |
| **Income** | 0.065 |
|  | (-0.022 - 0.152) |
| **Income square** | -0.004 |
|  | (-0.011 - 0.004) |
| **House Ownership** | -0.032 |
|  | (-0.111 - 0.047) |
| **Household Size** | 0.043*** |
|  | (0.019 - 0.067) |
| **Work** | -0.870*** |
|  | (-0.935 - -0.806) |
| **Retired** | -1.398*** |
|  | (-1.493 - -1.303) |
| **Constant** | 10.026*** |
|  | (8.421 - 11.631) |
|  |  |
| **Obs.** | 349,989 |
| **No. individuals** | 0.011 |

***Note***: A fixed-effects model is applied. 95% confidence interval is given in parentheses. Year dummies are included in all models, but their coefficients are not reported, for brevity.

****p*<0.001, ***p*<0.01, **p*<0.05

**Table A13: Psychological wellbeing and caring intensity by caregiver-care recipient relationship, with care-recipient’s health status**

|  | **(1)** | **(2)** | **(3)** | **(4)** | **(5)** |
| --- | --- | --- | --- | --- | --- |
|  | **Spouse** | **Child** | **Parent** | **Other relative** | **Non-relative** |
|  | **ß (95% CI)** | **ß (95% CI)** | **ß (95% CI)** | **ß (95% CI)** | **ß (95% CI)** |
|  |  |  |  |  |  |
| **0-4 hrs.** | 0.686*** | 0.467 | -0.064 | -0.203** | -0.200** |
|  | (0.428 - 0.944) | (-0.304 - 1.237) | (-0.151 - 0.023) | (-0.335 - -0.071) | (-0.322 - -0.078) |
| **5-9 hrs.** | 0.641*** | 0.551 | 0.199*** | 0.044 | -0.215 |
|  | (0.363 - 0.919) | (-0.300 - 1.403) | (0.089 - 0.309) | (-0.152 - 0.241) | (-0.448 - 0.017) |
| **10-19 hrs.** | 0.741*** | 0.687 | 0.437*** | 0.256 | -0.379* |
|  | (0.464 - 1.018) | (-0.185 - 1.559) | (0.295 - 0.578) | (-0.020 - 0.533) | (-0.733 - -0.025) |
| **20-34 hrs.** | 1.203*** | 0.576 | 0.679*** | 0.201 | -0.229 |
|  | (0.899 - 1.507) | (-0.349 - 1.502) | (0.456 - 0.901) | (-0.223 - 0.625) | (-0.813 - 0.354) |
| **35-49 hrs.** | 1.150*** | 1.101 | 0.434* | 0.384 | 0.120 |
|  | (0.761 - 1.539) | (-0.028 - 2.230) | (0.066 - 0.801) | (-0.296 - 1.064) | (-0.855 - 1.095) |
| **50+ hrs.** | 1.422*** | 1.245** | 0.666*** | -0.051 | -0.779 |
|  | (1.179 - 1.665) | (0.390 - 2.099) | (0.314 - 1.018) | (-0.706 - 0.603) | (-1.651 - 0.093) |
| **Excellent**  **(care-recipient)** | -1.063* | -1.477** | -0.573 | -2.598** | -0.163 |
|  | (-1.939 - -0.187) | (-2.583 - -0.370) | (-1.527 - 0.382) | (-4.437 - -0.759) | (-2.462 - 2.135) |
| **Good**  **(care-recipient)** | -0.238* | -0.639* | -0.047 | -0.547* | 0.152 |
|  | (-0.458 - -0.018) | (-1.275 - -0.004) | (-0.351 - 0.258) | (-1.049 - -0.045) | (-0.502 - 0.806) |
| **Fair**  **(care-recipient)** | -0.431*** | 0.166 | -0.247 | 0.962*** | 0.442 |
|  | (-0.626 - -0.237) | (-0.475 - 0.807) | (-0.509 - 0.014) | (0.512 - 1.413) | (-0.153 - 1.036) |
| **Degree** | 0.563*** | 0.562*** | 0.558*** | 0.532*** | 0.566*** |
|  | (0.425 - 0.701) | (0.423 - 0.700) | (0.423 - 0.693) | (0.396 - 0.668) | (0.429 - 0.703) |
| **Age** | 0.001 | 0.008 | 0.008 | 0.021 | 0.008 |
|  | (-0.055 - 0.057) | (-0.049 - 0.065) | (-0.047 - 0.062) | (-0.035 - 0.077) | (-0.049 - 0.064) |
| **Married** | 0.063 | 0.053 | 0.075 | 0.061 | 0.066 |
|  | (-0.018 - 0.144) | (-0.029 - 0.134) | (-0.004 - 0.154) | (-0.019 - 0.141) | (-0.015 - 0.146) |
| **Widowed** | 1.339*** | 1.309*** | 1.378*** | 1.289*** | 1.387*** |
|  | (1.048 - 1.629) | (1.009 - 1.609) | (1.091 - 1.665) | (0.992 - 1.585) | (1.092 - 1.683) |
| **Disabled** | 0.886*** | 0.886*** | 0.880*** | 0.886*** | 0.880*** |
|  | (0.831 - 0.942) | (0.830 - 0.943) | (0.827 - 0.933) | (0.831 - 0.942) | (0.825 - 0.936) |
| **Income** | 0.059 | 0.053 | 0.078 | 0.056 | 0.054 |
|  | (-0.028 - 0.146) | (-0.035 - 0.140) | (-0.006 - 0.162) | (-0.030 - 0.142) | (-0.032 - 0.140) |
| **Income square** | -0.003 | -0.002 | -0.004 | -0.003 | -0.002 |
|  | (-0.011 - 0.004) | (-0.010 - 0.005) | (-0.012 - 0.003) | (-0.010 - 0.005) | (-0.010 - 0.005) |
| **House Ownership** | -0.037 | -0.036 | -0.034 | -0.039 | -0.041 |
|  | (-0.116 - 0.043) | (-0.117 - 0.044) | (-0.112 - 0.044) | (-0.118 - 0.040) | (-0.121 - 0.038) |
| **Household Size** | 0.045*** | 0.044*** | 0.039** | 0.041** | 0.051*** |
|  | (0.021 - 0.069) | (0.019 - 0.068) | (0.015 - 0.063) | (0.016 - 0.065) | (0.027 - 0.076) |
| **Work** | -0.860*** | -0.862*** | -0.889*** | -0.853*** | -0.860*** |
|  | (-0.925 - -0.796) | (-0.928 - -0.797) | (-0.952 - -0.826) | (-0.917 - -0.789) | (-0.924 - -0.795) |
| **Retired** | -1.382*** | -1.413*** | -1.466*** | -1.387*** | -1.396*** |
|  | (-1.477 - -1.287) | (-1.511 - -1.315) | (-1.558 - -1.374) | (-1.484 - -1.291) | (-1.492 - -1.301) |
| **Constant** | 10.206*** | 10.020*** | 9.967*** | 9.639*** | 9.997*** |
|  | (8.591 - 11.821) | (8.408 - 11.632) | (8.414 - 11.520) | (8.053 - 11.225) | (8.394 - 11.601) |
|  |  |  |  |  |  |
| **Obs.** | 347,305 | 338,618 | 367,267 | 348,022 | 346,807 |
| **No. individuals** | 73,786 | 72,882 | 74,870 | 73,785 | 73,380 |

***Note***: A fixed-effects model is applied. 95% confidence interval is given in parentheses. Year dummies are included in all models, but their coefficients are not reported, for brevity.

****p*<0.001, ***p*<0.01, **p*<0.05

**Table A14: Psychological wellbeing and caregiver-care recipient relationship by care intensity, with care-recipient’s health status**

|  | **(1)** | **(2)** | **(3)** | **(4)** |
| --- | --- | --- | --- | --- |
|  | **< 10 hrs** | **[10 20)** | **[20 50)** | **≥ 50 hrs** |
|  | **ß (95% CI)** | **ß (95% CI)** | **ß (95% CI)** | **ß (95% CI)** |
|  |  |  |  |  |
| **Spouse** | 0.454*** | 0.434* | 0.978*** | 1.524*** |
|  | (0.220 - 0.688) | (0.069 - 0.798) | (0.627 - 1.329) | (1.226 - 1.823) |
| **Child** | 0.307 | 0.051 | 0.686 | 1.055* |
|  | (-0.224 - 0.837) | (-0.845 - 0.947) | (-0.134 - 1.507) | (0.141 - 1.968) |
| **Parent** | 0.031 | 0.395*** | 0.608*** | 0.632** |
|  | (-0.043 - 0.105) | (0.237 - 0.554) | (0.378 - 0.837) | (0.224 - 1.039) |
| **Other relative** | -0.117* | 0.149 | 0.072 | -0.283 |
|  | (-0.230 - -0.005) | (-0.151 - 0.448) | (-0.333 - 0.477) | (-0.948 - 0.382) |
| **Non-relative** | -0.186*** | -0.461* | -0.318 | -0.439 |
|  | (-0.295 - -0.076) | (-0.833 - -0.089) | (-0.848 - 0.213) | (-1.277 - 0.398) |
| **Excellent**  **(care-recipient)** | -0.981* | -1.745* | -0.278 | -1.181* |
|  | (-1.755 - -0.207) | (-3.356 - -0.133) | (-1.699 - 1.143) | (-2.339 - -0.023) |
| **Good**  **(care-recipient)** | -0.197 | -0.324 | -0.260 | -0.411* |
|  | (-0.440 - 0.046) | (-0.748 - 0.101) | (-0.697 - 0.177) | (-0.771 - -0.051) |
| **Fair**  **(care-recipient)** | -0.107 | 0.100 | -0.437* | -0.427** |
|  | (-0.336 - 0.122) | (-0.281 - 0.482) | (-0.820 - -0.055) | (-0.740 - -0.113) |
| **Degree** | 0.547*** | 0.554*** | 0.552*** | 0.568*** |
|  | (0.416 - 0.679) | (0.416 - 0.691) | (0.413 - 0.690) | (0.430 - 0.707) |
| **Age** | 0.015 | 0.004 | 0.017 | 0.004 |
|  | (-0.038 - 0.069) | (-0.053 - 0.060) | (-0.040 - 0.073) | (-0.053 - 0.061) |
| **Married** | 0.076 | 0.045 | 0.065 | 0.051 |
|  | (-0.001 - 0.154) | (-0.036 - 0.126) | (-0.016 - 0.146) | (-0.030 - 0.133) |
| **Widowed** | 1.360*** | 1.298*** | 1.391*** | 1.328*** |
|  | (1.082 - 1.639) | (1.000 - 1.596) | (1.094 - 1.688) | (1.034 - 1.623) |
| **Disabled** | 0.880*** | 0.877*** | 0.888*** | 0.882*** |
|  | (0.827 - 0.932) | (0.821 - 0.933) | (0.832 - 0.945) | (0.826 - 0.938) |
| **Income** | 0.069 | 0.062 | 0.058 | 0.061 |
|  | (-0.013 - 0.151) | (-0.025 - 0.149) | (-0.029 - 0.146) | (-0.026 - 0.148) |
| **Income square** | -0.004 | -0.003 | -0.003 | -0.003 |
|  | (-0.011 - 0.004) | (-0.011 - 0.004) | (-0.011 - 0.005) | (-0.011 - 0.004) |
| **House Ownership** | -0.042 | -0.033 | -0.033 | -0.026 |
|  | (-0.119 - 0.034) | (-0.113 - 0.047) | (-0.113 - 0.047) | (-0.106 - 0.054) |
| **Household Size** | 0.046*** | 0.043*** | 0.042*** | 0.042*** |
|  | (0.022 - 0.069) | (0.018 - 0.067) | (0.017 - 0.066) | (0.018 - 0.067) |
| **Work** | -0.883*** | -0.858*** | -0.865*** | -0.860*** |
|  | (-0.944 - -0.821) | (-0.923 - -0.793) | (-0.930 - -0.800) | (-0.925 - -0.795) |
| **Retired** | -1.435*** | -1.406*** | -1.414*** | -1.399*** |
|  | (-1.525 - -1.345) | (-1.502 - -1.309) | (-1.510 - -1.317) | (-1.496 - -1.302) |
| **Constant** | 9.748*** | 10.135*** | 9.768*** | 10.111*** |
|  | (8.210 - 11.287) | (8.532 - 11.737) | (8.160 - 11.375) | (8.495 - 11.727) |
|  |  |  |  |  |
| **Obs.** | 377,016 | 345,041 | 342,587 | 341,883 |
| **No. individuals** | 75,410 | 73,888 | 73,719 | 73,623 |

***Note***: A fixed-effects model is applied. 95% confidence interval is given in parentheses. Year dummies are included in all models, but their coefficients are not reported, for brevity.

****p*<0.001, ***p*<0.01, **p*<0.05

Figure A1: Trace plots of summaries of imputed GHQ-12

Figure A2: Trace plots of summaries of imputed Income

**Table A15: Psychological wellbeing and caring intensity by location of care provision with imputation**

|  | **(1)** | **(2)** |
| --- | --- | --- |
|  | **Co-residence** | **Extra-residence** |
|  | **ß (95% CI)** | **ß (95% CI)** |
|  |  |  |
| **0-4 hrs.** | 0.155* | -0.122*** |
|  | (0.002 - 0.309) | (-0.187 - -0.058) |
| **5-9 hrs.** | 0.289*** | 0.133** |
|  | (0.118 - 0.460) | (0.042 - 0.224) |
| **10-19 hrs.** | 0.476*** | 0.334*** |
|  | (0.307 - 0.645) | (0.210 - 0.458) |
| **20-34 hrs.** | 0.615*** | 0.553*** |
|  | (0.414 - 0.817) | (0.353 - 0.752) |
| **35-49 hrs.** | 0.569*** | 0.608*** |
|  | (0.325 - 0.814) | (0.285 - 0.932) |
| **50+ hrs.** | 1.026*** | 0.820*** |
|  | (0.879 - 1.172) | (0.386 - 1.254) |
| **Degree** | 0.592*** | 0.555*** |
|  | (0.458 - 0.726) | (0.426 - 0.684) |
| **Age** | 0.013 | 0.004 |
|  | (-0.044 - 0.069) | (-0.050 - 0.058) |
| **Married** | 0.153*** | 0.121** |
|  | (0.072 - 0.235) | (0.043 - 0.198) |
| **Widowed** | 1.176*** | 1.142*** |
|  | (0.891 - 1.461) | (0.892 - 1.391) |
| **Disabled** | 1.041*** | 0.998*** |
|  | (0.984 - 1.098) | (0.944 - 1.052) |
| **Income** | 0.058 | 0.065 |
|  | (-0.025 - 0.141) | (-0.015 - 0.146) |
| **Income square** | -0.004 | -0.004 |
|  | (-0.011 - 0.004) | (-0.011 - 0.003) |
| **House Ownership** | -0.079 | -0.051 |
|  | (-0.158 - 0.001) | (-0.128 - 0.027) |
| **Household Size** | 0.027* | 0.033** |
|  | (0.003 - 0.052) | (0.010 - 0.056) |
| **Work** | -0.911*** | -0.922*** |
|  | (-0.972 - -0.849) | (-0.981 - -0.864) |
| **Retired** | -1.479*** | -1.544*** |
|  | (-1.573 - -1.385) | (-1.631 - -1.456) |
| **Constant** | 9.958*** | 10.205*** |
|  | (8.373 - 11.543) | (8.662 - 11.747) |
|  |  |  |
| **Obs.** | 411,983 | 441,629 |
| **No. individuals** | 83,368 | 85,333 |

***Note***: A fixed-effects model is applied 95% confidence interval is given in parentheses. Year dummies are included in all models, but their coefficients are not reported, for brevity.

****p*<0.001, ***p*<0.01, **p*<0.05

**Table A16: Psychological wellbeing and caring intensity by caregiver-care recipient relationship with imputation**

|  | **(1)** | **(2)** | **(3)** | **(4)** | **(5)** |
| --- | --- | --- | --- | --- | --- |
|  | **Spouse** | **Child** | **Parent** | **Other relative** | **Non-relative** |
|  | **ß (95% CI)** | **ß (95% CI)** | **ß (95% CI)** | **ß (95% CI)** | **ß (95% CI)** |
|  |  |  |  |  |  |
| **0-4 hrs.** | 0.345*** | 0.048 | -0.062 | -0.143* | -0.183** |
|  | (0.144 - 0.545) | (-0.304 - 0.399) | (-0.149 - 0.025) | (-0.273 - -0.014) | (-0.307 - -0.060) |
| **5-9 hrs.** | 0.459*** | -0.016 | 0.225*** | 0.176 | -0.153 |
|  | (0.240 - 0.677) | (-0.403 - 0.371) | (0.112 - 0.338) | (-0.014 - 0.365) | (-0.384 - 0.077) |
| **10-19 hrs.** | 0.611*** | 0.292 | 0.424*** | 0.198 | -0.223 |
|  | (0.392 - 0.830) | (-0.050 - 0.634) | (0.288 - 0.561) | (-0.060 - 0.457) | (-0.573 - 0.127) |
| **20-34 hrs.** | 0.847*** | 0.323 | 0.643*** | 0.201 | -0.087 |
|  | (0.589 - 1.106) | (-0.045 - 0.690) | (0.434 - 0.852) | (-0.175 - 0.577) | (-0.655 - 0.481) |
| **35-49 hrs.** | 0.982*** | 0.352 | 0.387* | 0.611* | -0.020 |
|  | (0.656 - 1.308) | (-0.087 - 0.791) | (0.059 - 0.715) | (0.031 - 1.191) | (-0.893 - 0.854) |
| **50+ hrs.** | 1.293*** | 0.859*** | 0.751*** | 0.312 | -0.328 |
|  | (1.097 - 1.489) | (0.592 - 1.125) | (0.481 - 1.021) | (-0.185 - 0.810) | (-0.984 - 0.329) |
| **Degree** | 0.553*** | 0.561*** | 0.560*** | 0.554*** | 0.569*** |
|  | (0.417 - 0.688) | (0.425 - 0.696) | (0.428 - 0.692) | (0.422 - 0.686) | (0.434 - 0.703) |
| **Age** | 0.006 | 0.009 | 0.010 | 0.017 | 0.009 |
|  | (-0.051 - 0.064) | (-0.049 - 0.067) | (-0.046 - 0.066) | (-0.041 - 0.075) | (-0.049 - 0.067) |
| **Married** | 0.130** | 0.116** | 0.138*** | 0.126** | 0.135** |
|  | (0.048 - 0.213) | (0.032 - 0.199) | (0.058 - 0.219) | (0.044 - 0.208) | (0.053 - 0.218) |
| **Widowed** | 1.227*** | 1.205*** | 1.328*** | 1.178*** | 1.324*** |
|  | (0.921 - 1.534) | (0.900 - 1.509) | (1.032 - 1.624) | (0.870 - 1.486) | (1.014 - 1.635) |
| **Disable** | 1.026*** | 1.035*** | 1.017*** | 1.020*** | 1.020*** |
|  | (0.968 - 1.083) | (0.977 - 1.094) | (0.961 - 1.073) | (0.962 - 1.077) | (0.962 - 1.078) |
| **Income** | 0.055 | 0.049 | 0.063 | 0.056 | 0.048 |
|  | (-0.030 - 0.139) | (-0.036 - 0.134) | (-0.018 - 0.145) | (-0.027 - 0.139) | (-0.036 - 0.133) |
| **Income square** | -0.003 | -0.003 | -0.004 | -0.004 | -0.003 |
|  | (-0.011 - 0.004) | (-0.011 - 0.005) | (-0.011 - 0.003) | (-0.011 - 0.004) | (-0.010 - 0.005) |
| **House Ownership** | -0.077 | -0.079 | -0.071 | -0.080 | -0.078 |
|  | (-0.158 - 0.004) | (-0.161 - 0.003) | (-0.150 - 0.009) | (-0.160 - 0.001) | (-0.159 - 0.003) |
| **Household Size** | 0.034** | 0.027* | 0.030* | 0.029* | 0.037** |
|  | (0.010 - 0.059) | (0.002 - 0.052) | (0.006 - 0.054) | (0.004 - 0.053) | (0.012 - 0.062) |
| **Work** | -0.904*** | -0.903*** | -0.915*** | -0.892*** | -0.901*** |
|  | (-0.967 - -0.842) | (-0.965 - -0.841) | (-0.976 - -0.855) | (-0.953 - -0.830) | (-0.963 - -0.839) |
| **Retired** | -1.471*** | -1.520*** | -1.545*** | -1.489*** | -1.499*** |
|  | (-1.566 - -1.377) | (-1.619 - -1.422) | (-1.637 - -1.453) | (-1.586 - -1.392) | (-1.595 - -1.403) |
| **Constant** | 10.124*** | 10.105*** | 10.023*** | 9.837*** | 10.060*** |
|  | (8.513 - 11.735) | (8.508 - 11.701) | (8.471 - 11.575) | (8.255 - 11.419) | (8.462 - 11.657) |
|  |  |  |  |  |  |
| **Obs.** | 400,370 | 392,556 | 421,909 | 399,071 | 396,264 |
| **No. individuals** | 81,818 | 81,005 | 82,884 | 81,689 | 81,050 |

*Note*: A fixed-effects model is applied. 95% confidence interval is given in parentheses. Year dummies are included in all models, but their coefficients are not reported, for brevity.

****p*<0.001, ***p*<0.01, **p*<0.05

**Table A17: Psychological wellbeing and caring location by caring intensity with imputation**

|  | **(1)** | **(2)** | **(3)** | **(4)** |
| --- | --- | --- | --- | --- |
|  | **< 10 hrs** | **[10 20)** | **[20 50)** | ≥ **50 hrs** |
|  | **ß (95% CI)** | **ß (95% CI)** | **ß (95% CI)** | **ß (95% CI)** |
|  |  |  |  |  |
| **Extra-residence** | -0.054 | 0.372*** | 0.598*** | 1.061*** |
|  | (-0.116 - 0.007) | (0.222 - 0.521) | (0.374 - 0.821) | (0.502 - 1.620) |
| **Co-residence** | 0.175** | 0.409*** | 0.560*** | 1.067*** |
|  | (0.044 - 0.305) | (0.207 - 0.611) | (0.364 - 0.756) | (0.896 - 1.239) |
| **Degree** | 0.562*** | 0.544*** | 0.537*** | 0.553*** |
|  | (0.424 - 0.700) | (0.400 - 0.689) | (0.392 - 0.682) | (0.408 - 0.699) |
| **Age** | 0.015 | 0.003 | 0.014 | 0.004 |
|  | (-0.042 - 0.073) | (-0.058 - 0.063) | (-0.047 - 0.075) | (-0.057 - 0.066) |
| **Married** | 0.082 | 0.051 | 0.070 | 0.066 |
|  | (-0.000 - 0.163) | (-0.034 - 0.136) | (-0.015 - 0.156) | (-0.019 - 0.151) |
| **Widowed** | 1.284*** | 1.174*** | 1.240*** | 1.165*** |
|  | (0.988 - 1.580) | (0.855 - 1.492) | (0.927 - 1.553) | (0.860 - 1.471) |
| **Disabled** | 1.000*** | 1.005*** | 1.012*** | 1.008*** |
|  | (0.944 - 1.055) | (0.945 - 1.065) | (0.952 - 1.072) | (0.948 - 1.067) |
| **Income** | 0.075 | 0.058 | 0.061 | 0.060 |
|  | (-0.010 - 0.160) | (-0.032 - 0.147) | (-0.029 - 0.151) | (-0.031 - 0.150) |
| **Income square** | -0.004 | -0.003 | -0.003 | -0.003 |
|  | (-0.012 - 0.003) | (-0.011 - 0.005) | (-0.011 - 0.005) | (-0.011 - 0.005) |
| **House ownership** | -0.063 | -0.065 | -0.068 | -0.061 |
|  | (-0.142 - 0.016) | (-0.148 - 0.018) | (-0.151 - 0.015) | (-0.144 - 0.022) |
| **Household size** | 0.036** | 0.033* | 0.032* | 0.031* |
|  | (0.011 - 0.061) | (0.007 - 0.059) | (0.006 - 0.058) | (0.004 - 0.057) |
| **Work** | -0.914*** | -0.893*** | -0.901*** | -0.901*** |
|  | (-0.978 - -0.850) | (-0.961 - -0.826) | (-0.968 - -0.833) | (-0.969 - -0.834) |
| **Retired** | -1.532*** | -1.494*** | -1.510*** | -1.483*** |
|  | (-1.624 - -1.439) | (-1.594 - -1.395) | (-1.611 - -1.409) | (-1.583 - -1.383) |
| **Constant** | 9.825*** | 10.284*** | 9.950*** | 10.232*** |
|  | (8.170 - 11.481) | (8.572 - 11.995) | (8.231 - 11.669) | (8.490 - 11.973) |
|  |  |  |  |  |
| **Obs.** | 405,224 | 371,125 | 369,175 | 370,912 |
| **No. individuals** | 79,296 | 77,733 | 77,738 | 77,933 |

*Note*: A fixed-effects model is applied. 95% confidence interval is given in parentheses. Year dummies are included in all models, but their coefficients are not reported, for brevity.

****p*<0.001, ***p*<0.01, **p*<0.05

**Table A18: Psychological wellbeing and caregiver-care recipient relationship by care intensity with imputation**

|  | **(1)** | **(2)** | **(3)** | **(4)** |
| --- | --- | --- | --- | --- |
|  | **< 10 hrs** | **[10 20)** | **[20 50)** | **>= 50 hrs** |
|  | **ß (95% CI)** | **ß (95% CI)** | **ß (95% CI)** | **ß (95% CI)** |
|  |  |  |  |  |
| **Spouse** | 0.324*** | 0.580*** | 0.758*** | 1.373*** |
|  | (0.157 - 0.491) | (0.327 - 0.834) | (0.498 - 1.018) | (1.148 - 1.598) |
| **Child** | 0.125 | 0.031 | 0.355 | 0.778*** |
|  | (-0.175 - 0.426) | (-0.373 - 0.434) | (-0.011 - 0.720) | (0.468 - 1.089) |
| **Parent** | 0.045 | 0.394*** | 0.587*** | 0.487** |
|  | (-0.030 - 0.120) | (0.235 - 0.553) | (0.370 - 0.803) | (0.164 - 0.811) |
| **Other relative** | -0.115 | 0.129 | 0.094 | 0.046 |
|  | (-0.230 - 0.001) | (-0.163 - 0.420) | (-0.280 - 0.469) | (-0.439 - 0.531) |
| **Non-relative** | -0.160** | -0.342 | -0.379 | -0.545 |
|  | (-0.273 - -0.046) | (-0.719 - 0.035) | (-0.866 - 0.108) | (-1.181 - 0.091) |
| **Degree** | 0.563*** | 0.547*** | 0.534*** | 0.556*** |
|  | (0.424 - 0.701) | (0.402 - 0.691) | (0.389 - 0.679) | (0.411 - 0.702) |
| **Age** | 0.016 | 0.004 | 0.013 | 0.003 |
|  | (-0.041 - 0.074) | (-0.056 - 0.065) | (-0.048 - 0.074) | (-0.058 - 0.064) |
| **Married** | 0.078 | 0.049 | 0.070 | 0.057 |
|  | (-0.003 - 0.160) | (-0.036 - 0.134) | (-0.015 - 0.155) | (-0.028 - 0.141) |
| **Widowed** | 1.260*** | 1.166*** | 1.271*** | 1.165*** |
|  | (0.965 - 1.555) | (0.848 - 1.485) | (0.958 - 1.584) | (0.859 - 1.471) |
| **Disabled** | 0.998*** | 1.004*** | 1.015*** | 1.009*** |
|  | (0.943 - 1.054) | (0.945 - 1.064) | (0.955 - 1.075) | (0.949 - 1.068) |
| **Income** | 0.073 | 0.061 | 0.061 | 0.058 |
|  | (-0.012 - 0.157) | (-0.028 - 0.150) | (-0.029 - 0.150) | (-0.032 - 0.148) |
| **Income square** | -0.004 | -0.003 | -0.003 | -0.003 |
|  | (-0.012 - 0.003) | (-0.011 - 0.005) | (-0.011 - 0.005) | (-0.011 - 0.005) |
| **House ownership** | -0.065 | -0.065 | -0.068 | -0.060 |
|  | (-0.144 - 0.014) | (-0.148 - 0.017) | (-0.151 - 0.015) | (-0.143 - 0.023) |
| **Household size** | 0.036** | 0.033* | 0.031* | 0.032* |
|  | (0.012 - 0.061) | (0.007 - 0.058) | (0.005 - 0.057) | (0.006 - 0.059) |
| **Work** | -0.918*** | -0.893*** | -0.901*** | -0.900*** |
|  | (-0.982 - -0.855) | (-0.960 - -0.826) | (-0.968 - -0.834) | (-0.967 - -0.833) |
| **Retired** | -1.532*** | -1.491*** | -1.511*** | -1.491*** |
|  | (-1.624 - -1.440) | (-1.590 - -1.391) | (-1.611 - -1.410) | (-1.590 - -1.392) |
| **Constant** | 9.823*** | 10.242*** | 9.981*** | 10.293*** |
|  | (8.170 - 11.477) | (8.533 - 11.951) | (8.262 - 11.699) | (8.556 - 12.030) |
|  |  |  |  |  |
| **Obs.** | 407,018 | 371,796 | 369,905 | 371,660 |
| **No. individuals** | 79,532 | 77,883 | 77,891 | 78,067 |

*Note*: A fixed-effects model is applied. 95% confidence interval is given in parentheses. Year dummies are included in all models, but their coefficients are not reported, for brevity.

****p*<0.001, ***p*<0.01, **p*<0.05

**Table A19: Psychological wellbeing and caring intensity by location of care provision with bootstrap standard error**

|  | **(1)** | **(2)** |
| --- | --- | --- |
|  | **Co-residence** | **Extra-residence** |
|  | **ß (95% CI)** | **ß (95% CI)** |
|  |  |  |
| **0-4 hrs.** | 0.146 | -0.132*** |
|  | (-0.035 - 0.326) | (-0.198 - -0.066) |
| **5-9 hrs.** | 0.262** | 0.102* |
|  | (0.089 - 0.436) | (0.001 - 0.203) |
| **10-19 hrs.** | 0.398*** | 0.294*** |
|  | (0.207 - 0.589) | (0.149 - 0.440) |
| **20-34 hrs.** | 0.617*** | 0.541*** |
|  | (0.385 - 0.849) | (0.300 - 0.783) |
| **35-49 hrs.** | 0.528*** | 0.573** |
|  | (0.238 - 0.818) | (0.182 - 0.965) |
| **50+ hrs.** | 0.949*** | 0.841*** |
|  | (0.754 - 1.145) | (0.348 - 1.335) |
| **Degree** | 0.605*** | 0.551*** |
|  | (0.416 - 0.795) | (0.367 - 0.736) |
| **Age** | 0.013 | 0.003 |
|  | (-0.050 - 0.076) | (-0.062 - 0.069) |
| **Married** | 0.088 | 0.056 |
|  | (-0.036 - 0.211) | (-0.065 - 0.177) |
| **Widowed** | 1.243*** | 1.146*** |
|  | (0.857 - 1.629) | (0.811 - 1.481) |
| **Disabled** | 0.901*** | 0.870*** |
|  | (0.832 - 0.970) | (0.808 - 0.933) |
| **Income** | 0.063 | 0.071 |
|  | (-0.030 - 0.156) | (-0.013 - 0.155) |
| **Income square** | -0.004 | -0.004 |
|  | (-0.012 - 0.005) | (-0.011 - 0.004) |
| **House Ownership** | -0.036 | -0.016 |
|  | (-0.142 - 0.070) | (-0.119 - 0.088) |
| **Household Size** | 0.038* | 0.043** |
|  | (0.008 - 0.069) | (0.014 - 0.073) |
| **Work** | -0.868*** | -0.894*** |
|  | (-0.957 - -0.779) | (-0.973 - -0.815) |
| **Retired** | -1.368*** | -1.458*** |
|  | (-1.487 - -1.249) | (-1.571 - -1.345) |
| **Constant** | 9.874*** | 10.147*** |
|  | (8.047 - 11.700) | (8.237 - 12.056) |
|  |  |  |
| **Obs.** | 360,204 | 389,013 |
| **No. individuals** | 75,705 | 77,707 |

*Note*: A fixed-effects model is applied. 95% confidence interval with bootstrap standard error is given in parentheses. Year dummies are included in all models, but their coefficients are not reported, for brevity.

****p*<0.001, ***p*<0.01, **p*<0.05

**Table A20: Psychological wellbeing and caring intensity by caregiver-care recipient relationship with bootstrap standard error**

|  | **(1)** | **(2)** | **(3)** | **(4)** | **(5)** |
| --- | --- | --- | --- | --- | --- |
|  | **Spouse** | **Child** | **Parent** | **Other relative** | **Non-relative** |
|  | **ß (95% CI)** | **ß (95% CI)** | **ß (95% CI)** | **ß (95% CI)** | **ß (95% CI)** |
|  |  |  |  |  |  |
| **0-4 hrs.** | 0.396*** | 0.037 | -0.071 | -0.180** | -0.183** |
|  | (0.195 - 0.597) | (-0.313 - 0.386) | (-0.166 - 0.024) | (-0.302 - -0.059) | (-0.301 - -0.065) |
| **5-9 hrs.** | 0.386*** | 0.089 | 0.201*** | 0.061 | -0.210 |
|  | (0.178 - 0.593) | (-0.349 - 0.528) | (0.083 - 0.319) | (-0.148 - 0.270) | (-0.427 - 0.007) |
| **10-19 hrs.** | 0.588*** | 0.288 | 0.377*** | 0.118 | -0.289 |
|  | (0.373 - 0.802) | (-0.111 - 0.686) | (0.232 - 0.522) | (-0.208 - 0.444) | (-0.670 - 0.091) |
| **20-34 hrs.** | 0.955*** | 0.322 | 0.641*** | 0.139 | -0.286 |
|  | (0.672 - 1.238) | (-0.101 - 0.745) | (0.388 - 0.894) | (-0.294 - 0.572) | (-0.975 - 0.403) |
| **35-49 hrs.** | 0.967*** | 0.188 | 0.351* | 0.483 | -0.065 |
|  | (0.606 - 1.329) | (-0.346 - 0.721) | (0.003 - 0.700) | (-0.243 - 1.209) | (-0.842 - 0.712) |
| **50+ hrs.** | 1.273*** | 0.692*** | 0.692*** | 0.189 | -0.446 |
|  | (1.044 - 1.502) | (0.326 - 1.058) | (0.350 - 1.035) | (-0.456 - 0.835) | (-1.249 - 0.357) |
| **Degree** | 0.561*** | 0.569*** | 0.564*** | 0.553*** | 0.569*** |
|  | (0.386 - 0.735) | (0.393 - 0.746) | (0.383 - 0.745) | (0.369 - 0.738) | (0.387 - 0.752) |
| **Age** | 0.003 | 0.009 | 0.008 | 0.022 | 0.009 |
|  | (-0.061 - 0.067) | (-0.061 - 0.080) | (-0.059 - 0.074) | (-0.042 - 0.087) | (-0.058 - 0.076) |
| **Married** | 0.056 | 0.045 | 0.071 | 0.053 | 0.064 |
|  | (-0.065 - 0.177) | (-0.079 - 0.169) | (-0.051 - 0.192) | (-0.062 - 0.168) | (-0.053 - 0.180) |
| **Widowed** | 1.286*** | 1.254*** | 1.378*** | 1.231*** | 1.388*** |
|  | (0.850 - 1.722) | (0.815 - 1.693) | (0.957 - 1.798) | (0.827 - 1.635) | (0.948 - 1.828) |
| **Disable** | 0.888*** | 0.899*** | 0.882*** | 0.886*** | 0.881*** |
|  | (0.819 - 0.956) | (0.832 - 0.966) | (0.814 - 0.950) | (0.818 - 0.954) | (0.810 - 0.953) |
| **Income** | 0.057 | 0.054 | 0.074 | 0.056 | 0.054 |
|  | (-0.031 - 0.145) | (-0.044 - 0.152) | (-0.009 - 0.158) | (-0.039 - 0.150) | (-0.051 - 0.159) |
| **Income square** | -0.003 | -0.003 | -0.004 | -0.003 | -0.003 |
|  | (-0.011 - 0.005) | (-0.011 - 0.006) | (-0.012 - 0.004) | (-0.011 - 0.006) | (-0.012 - 0.007) |
| **House Ownership** | -0.034 | -0.039 | -0.033 | -0.042 | -0.041 |
|  | (-0.136 - 0.068) | (-0.148 - 0.070) | (-0.133 - 0.067) | (-0.148 - 0.063) | (-0.146 - 0.064) |
| **Household Size** | 0.045** | 0.037* | 0.040* | 0.038** | 0.051** |
|  | (0.011 - 0.078) | (0.005 - 0.070) | (0.009 - 0.072) | (0.009 - 0.067) | (0.016 - 0.085) |
| **Work** | -0.864*** | -0.865*** | -0.889*** | -0.850*** | -0.863*** |
|  | (-0.946 - -0.781) | (-0.947 - -0.782) | (-0.973 - -0.804) | (-0.938 - -0.761) | (-0.943 - -0.782) |
| **Retired** | -1.366*** | -1.421*** | -1.462*** | -1.384*** | -1.396*** |
|  | (-1.489 - -1.242) | (-1.553 - -1.289) | (-1.577 - -1.348) | (-1.501 - -1.266) | (-1.512 - -1.281) |
| **Constant** | 10.154*** | 10.019*** | 9.994*** | 9.620*** | 9.962*** |
|  | (8.327 - 11.980) | (8.062 - 11.975) | (8.144 - 11.843) | (7.774 - 11.466) | (8.036 - 11.888) |
|  |  |  |  |  |  |
| **Obs.** | 350,488 | 343,745 | 370,432 | 349,450 | 347,352 |
| **No. individuals** | 74,276 | 73,461 | 75,298 | 74,113 | 73,520 |

*Note*: A fixed-effects model is applied. 95% confidence interval with bootstrap standard error is given in parentheses. Year dummies are included in all models, but their coefficients are not reported, for brevity.

****p*<0.001, ***p*<0.01, **p*<0.05

**Table A21: Psychological wellbeing and caring location by caring intensity with bootstrap standard error**

|  | **(1)** | **(2)** | **(3)** | **(4)** |
| --- | --- | --- | --- | --- |
|  | **< 10 hrs** | **[10 20)** | **[20 50)** | ≥ **50 hrs** |
|  | **ß (95% CI)** | **ß (95% CI)** | **ß (95% CI)** | **ß (95% CI)** |
|  |  |  |  |  |
| **Extra-residence** | -0.070* | 0.310*** | 0.552*** | 1.083** |
|  | (-0.136 - -0.005) | (0.136 - 0.484) | (0.292 - 0.812) | (0.353 - 1.813) |
| **Co-residence** | 0.155* | 0.271** | 0.483*** | 1.023*** |
|  | (0.006 - 0.304) | (0.068 - 0.473) | (0.244 - 0.722) | (0.782 - 1.264) |
| **Degree** | 0.569*** | 0.560*** | 0.552*** | 0.570*** |
|  | (0.384 - 0.753) | (0.363 - 0.756) | (0.371 - 0.733) | (0.383 - 0.757) |
| **Age** | 0.018 | 0.004 | 0.017 | 0.006 |
|  | (-0.046 - 0.083) | (-0.056 - 0.064) | (-0.044 - 0.078) | (-0.059 - 0.071) |
| **Married** | 0.077 | 0.044 | 0.067 | 0.061 |
|  | (-0.042 - 0.195) | (-0.066 - 0.154) | (-0.053 - 0.187) | (-0.070 - 0.192) |
| **Widowed** | 1.380*** | 1.258*** | 1.352*** | 1.287*** |
|  | (0.951 - 1.809) | (0.851 - 1.665) | (0.917 - 1.787) | (0.885 - 1.690) |
| **Disabled** | 0.881*** | 0.883*** | 0.890*** | 0.887*** |
|  | (0.817 - 0.945) | (0.812 - 0.953) | (0.826 - 0.954) | (0.820 - 0.953) |
| **Income** | 0.068 | 0.056 | 0.058 | 0.059 |
|  | (-0.024 - 0.161) | (-0.040 - 0.153) | (-0.037 - 0.152) | (-0.045 - 0.163) |
| **Income square** | -0.004 | -0.003 | -0.003 | -0.003 |
|  | (-0.012 - 0.005) | (-0.012 - 0.006) | (-0.011 - 0.006) | (-0.012 - 0.006) |
| **House ownership** | -0.039 | -0.035 | -0.040 | -0.029 |
|  | (-0.154 - 0.076) | (-0.140 - 0.069) | (-0.149 - 0.069) | (-0.140 - 0.082) |
| **Household size** | 0.046** | 0.041** | 0.040* | 0.039* |
|  | (0.016 - 0.075) | (0.012 - 0.071) | (0.008 - 0.071) | (0.008 - 0.071) |
| **Work** | -0.878*** | -0.857*** | -0.866*** | -0.862*** |
|  | (-0.965 - -0.790) | (-0.946 - -0.767) | (-0.952 - -0.780) | (-0.947 - -0.777) |
| **Retired** | -1.437*** | -1.397*** | -1.412*** | -1.376*** |
|  | (-1.551 - -1.323) | (-1.514 - -1.280) | (-1.528 - -1.295) | (-1.492 - -1.260) |
| **Constant** | 9.654*** | 10.143*** | 9.770*** | 10.082*** |
|  | (7.798 - 11.510) | (8.449 - 11.838) | (8.006 - 11.535) | (8.291 - 11.873) |
|  |  |  |  |  |
| **Obs.** | 378,704 | 346,084 | 344,156 | 345,566 |
| **No. individuals** | 75,729 | 74,148 | 74,137 | 74,289 |

*Note*: A fixed-effects model is applied. 95% confidence interval with bootstrap standard error is given in parentheses. Year dummies are included in all models, but their coefficients are not reported, for brevity.

****p*<0.001, ***p*<0.01, **p*<0.05

**Table A22: Psychological wellbeing and caregiver-care recipient relationship by care intensity with bootstrap standard error**

|  | **(1)** | **(2)** | **(3)** | **(4)** |
| --- | --- | --- | --- | --- |
|  | **< 10 hrs** | **[10 20)** | **[20 50)** | **>= 50 hrs** |
|  | **ß (95% CI)** | **ß (95% CI)** | **ß (95% CI)** | **ß (95% CI)** |
|  |  |  |  |  |
| **Spouse** | 0.321*** | 0.475*** | 0.781*** | 1.397*** |
|  | (0.159 - 0.484) | (0.212 - 0.737) | (0.497 - 1.064) | (1.138 - 1.656) |
| **Child** | 0.115 | -0.001 | 0.206 | 0.654** |
|  | (-0.227 - 0.458) | (-0.470 - 0.469) | (-0.287 - 0.699) | (0.255 - 1.054) |
| **Parent** | 0.028 | 0.334*** | 0.531*** | 0.459* |
|  | (-0.056 - 0.113) | (0.155 - 0.512) | (0.258 - 0.805) | (0.034 - 0.884) |
| **Other relative** | -0.127* | 0.053 | 0.030 | -0.006 |
|  | (-0.248 - -0.005) | (-0.244 - 0.349) | (-0.469 - 0.528) | (-0.569 - 0.556) |
| **Non-relative** | -0.187** | -0.395 | -0.558 | -0.565 |
|  | (-0.300 - -0.073) | (-0.832 - 0.043) | (-1.125 - 0.008) | (-1.255 - 0.124) |
| **Degree** | 0.569*** | 0.562*** | 0.551*** | 0.573*** |
|  | (0.377 - 0.760) | (0.375 - 0.749) | (0.364 - 0.737) | (0.400 - 0.747) |
| **Age** | 0.018 | 0.006 | 0.015 | 0.004 |
|  | (-0.044 - 0.081) | (-0.062 - 0.074) | (-0.050 - 0.080) | (-0.060 - 0.067) |
| **Married** | 0.073 | 0.043 | 0.067 | 0.051 |
|  | (-0.045 - 0.191) | (-0.085 - 0.170) | (-0.051 - 0.184) | (-0.068 - 0.170) |
| **Widowed** | 1.349*** | 1.250*** | 1.389*** | 1.288*** |
|  | (0.900 - 1.799) | (0.831 - 1.670) | (0.961 - 1.817) | (0.871 - 1.704) |
| **Disabled** | 0.881*** | 0.881*** | 0.892*** | 0.887*** |
|  | (0.816 - 0.946) | (0.811 - 0.951) | (0.826 - 0.958) | (0.818 - 0.957) |
| **Income** | 0.067 | 0.060 | 0.058 | 0.057 |
|  | (-0.028 - 0.161) | (-0.029 - 0.148) | (-0.037 - 0.152) | (-0.040 - 0.154) |
| **Income square** | -0.003 | -0.003 | -0.003 | -0.003 |
|  | (-0.012 - 0.005) | (-0.011 - 0.004) | (-0.012 - 0.006) | (-0.012 - 0.005) |
| **House ownership** | -0.042 | -0.035 | -0.039 | -0.028 |
|  | (-0.149 - 0.066) | (-0.146 - 0.075) | (-0.144 - 0.065) | (-0.128 - 0.073) |
| **Household size** | 0.046** | 0.041* | 0.039** | 0.041** |
|  | (0.014 - 0.078) | (0.009 - 0.073) | (0.010 - 0.068) | (0.012 - 0.071) |
| **Work** | -0.882*** | -0.855*** | -0.866*** | -0.861*** |
|  | (-0.964 - -0.800) | (-0.941 - -0.770) | (-0.950 - -0.782) | (-0.949 - -0.774) |
| **Retired** | -1.437*** | -1.392*** | -1.413*** | -1.384*** |
|  | (-1.554 - -1.319) | (-1.509 - -1.274) | (-1.529 - -1.298) | (-1.498 - -1.271) |
| **Constant** | 9.673*** | 10.092*** | 9.825*** | 10.157*** |
|  | (7.859 - 11.486) | (8.159 - 12.024) | (8.014 - 11.636) | (8.318 - 11.996) |
|  |  |  |  |  |
| **Obs.** | 380,403 | 346,712 | 344,826 | 346,268 |
| **No. individuals** | 75,967 | 74,297 | 74,288 | 74,429 |

*Note*: A fixed-effects model is applied. 95% confidence interval with bootstrap standard error is given in parentheses. Year dummies are included in all models, but their coefficients are not reported, for brevity.

****p*<0.001, ***p*<0.01, **p*<0.05

**Table A23: Psychological wellbeing and caring intensity by location of care provision**

|  | **(1)** | **(2)** |
| --- | --- | --- |
|  | **Co-residence** | **Extra-residence** |
|  | **ß (*p*-value)** | **ß (*p*-value)** |
|  |  |  |
| **0-4 hrs.** | 0.146 | -0.132^+^* |
|  | (0.063) | (0.000) |
| **5-9 hrs.** | 0.262^+^* | 0.102* |
|  | (0.003) | (0.024) |
| **10-19 hrs.** | 0.398^+^* | 0.294*** |
|  | (0.000) | (0.000) |
| **20-34 hrs.** | 0.617^+^* | 0.541*** |
|  | (0.000) | (0.000) |
| **35-49 hrs.** | 0.528^+^* | 0.573*** |
|  | (0.000) | (0.000) |
| **50+ hrs.** | 0.949^+^* | 0.841*** |
|  | (0.000) | (0.000) |
|  |  |  |
| **Observations** | 360,204 | 389,013 |
| **No. individuals** | 75,705 | 77,707 |

*Note*: A fixed-effects model is applied, which controls for educational attainment, age, marital status, disability, financial and employment status, and year dummies. The overall target significance level is set at 5%. Significance is indicated by '+' for *p*-values less than the Bonferroni-corrected threshold of 0.004 and by '*' for *p*-values equal to or less than the Benjamini-Hochberg adjusted threshold of 0.024.

**Table A24: Psychological wellbeing and caring intensity by caregiver-care recipient relationship**

|  | **(1)** | **(2)** | **(3)** | **(4)** | **(5)** |
| --- | --- | --- | --- | --- | --- |
|  | **Spouse** | **Child** | **Parent** | **Other relative** | **Non-relative** |
|  | **ß (*p*-value)** | **ß (*p*-value)** | **ß (*p*-value)** | **ß (*p*-value)** | **ß (*p*-value)** |
|  |  |  |  |  |  |
| **0-4 hrs.** | 0.396^+^* | 0.037 | -0.071 | -0.180* | -0.183* |
|  | (0.000) | (0.831) | (0.100) | (0.006) | (0.003) |
| **5-9 hrs.** | 0.386^+^* | 0.089 | 0.201^+^* | 0.061 | -0.210 |
|  | (0.001) | (0.635) | (0.000) | (0.524) | (0.070) |
| **10-19 hrs.** | 0.588^+^* | 0.288 | 0.377^+^* | 0.118 | -0.289 |
|  | (0.000) | (0.099) | (0.000) | (0.365) | (0.093) |
| **20-34 hrs.** | 0.955^+^* | 0.322 | 0.641^+^* | 0.139 | -0.286 |
|  | (0.000) | (0.082) | (0.000) | (0.466) | (0.294) |
| **35-49 hrs.** | 0.967^+^* | 0.188 | 0.351 | 0.483 | -0.065 |
|  | (0.000) | (0.386) | (0.031) | (0.103) | (0.881) |
| **50+ hrs.** | 1.273^+^* | 0.692^+^* | 0.692^+^* | 0.189 | -0.446 |
|  | (0.000) | (0.000) | (0.000) | (0.427) | (0.177) |
|  |  |  |  |  |  |
| **Observations** | 350,488 | 343,745 | 370,432 | 349,450 | 347,352 |
| **No. individuals** | 74,276 | 73,461 | 75,298 | 74,113 | 73,520 |

*Note*: A fixed-effects model is applied, which controls for educational attainment, age, marital status, disability, financial and employment status, and year dummies. The overall target significance level is set at 5%. Significance is indicated by '+' for *p*-values less than the Bonferroni-corrected threshold of 0.002 and by '*' for *p*-values equal to or less than the Benjamini-Hochberg adjusted threshold of 0.006.

**Table A25: Psychological wellbeing and caring location by caring intensity**

|  | **(1)** | **(2)** | **(3)** | **(4)** |
| --- | --- | --- | --- | --- |
|  | **< 10 hrs** | **[10 20)** | **[20 50)** | ≥ **50 hrs** |
|  | **ß (*p*-value)** | **ß (*p*-value)** | **ß (*p*-value)** | **ß (*p*-value)** |
|  |  |  |  |  |
| **Extra-residence** | -0.070* | 0.310^+^* | 0.552^+^* | 1.083^+^* |
|  | (0.018) | (0.000) | (0.000) | (0.000) |
| **Co-residence** | 0.155* | 0.271^+^* | 0.483^+^* | 1.023^+^* |
|  | (0.015) | (0.006) | (0.000) | (0.000) |
|  |  |  |  |  |
| **Obs.** | 378,704 | 346,084 | 344,156 | 345,566 |
| **No. individuals** | 75,729 | 74,148 | 74,137 | 74,289 |

*Note*: A fixed-effects model is applied, which controls for educational attainment, age, marital status, disability, financial and employment status, and year dummies. The overall target significance level is set at 5%. Significance is indicated by '+' for *p*-values less than the Bonferroni-corrected threshold of 0.006 and by '*' for *p*-values equal to or less than the Benjamini-Hochberg adjusted threshold of 0.018.

**Table A26: Psychological wellbeing and caregiver-care recipient relationship by care intensity**

|  | **(1)** | **(2)** | **(3)** | **(4)** |
| --- | --- | --- | --- | --- |
|  | **< 10 hrs** | **[10 20)** | **[20 50)** | **>= 50 hrs** |
|  | **ß (*p*-value)** | **ß (*p*-value)** | **ß (*p*-value)** | **ß (*p*-value)** |
|  |  |  |  |  |
| **Spouse** | 0.321^+^* | 0.475^+^* | 0.781^+^* | 1.397^+^* |
|  | (0.000) | (0.000) | (0.000) | (0.000) |
| **Child** | 0.115 | -0.001 | 0.206 | 0.654^+^* |
|  | (0.423) | (0.998) | (0.264) | (0.000) |
| **Parent** | 0.028 | 0.334^+^* | 0.531^+^* | 0.459* |
|  | (0.443) | (0.000) | (0.000) | (0.004) |
| **Other relative** | -0.127* | 0.053 | 0.030 | -0.006 |
|  | (0.024) | (0.714) | (0.871) | (0.978) |
| **Non-relative** | -0.187^+^* | -0.395 | -0.558* | -0.565 |
|  | (0.001) | (0.031) | (0.023) | (0.067) |
|  |  |  |  |  |
| **Obs.** | 380,403 | 346,712 | 344,826 | 346,268 |
| **No. individuals** | 75,967 | 74,297 | 74,288 | 74,429 |

*Note*: A fixed-effects model is applied, which controls for educational attainment, age, marital status, disability, financial and employment status, and year dummies. The overall target significance level is set at 5%. Significance is indicated by '+' for *p*-values less than the Bonferroni-corrected threshold of 0.003 and by '*' for *p*-values equal to or less than the Benjamini-Hochberg adjusted threshold of 0.024.

**References**

Bhaskaran, K., & Smeeth, L. (2014). What is the difference between missing completely at random and missing at random? *Int J Epidemiol*; 43(4):1336-9. doi: 10.1093/ije/dyu080. Epub 2014 Apr 4. PMID: 24706730; PMCID: PMC4121561.

Bonferroni, C. E. (1936). Teoria statistica delle classi e calcolo delle probabilit `a. Pubblicazioni del R Istituto Superiore di Scienze Economiche e Commerciali di Firenze 8:3–62

Efron, B., & Hastie, T. (2013). Computer age statistical inference, Student ed.; Cambridge University Press: Cambridge, UK.

Haynes, W. (2013). Benjamini–Hochberg Method. In: Dubitzky, W., Wolkenhauer, O., Cho, KH., Yokota, H. (eds) Encyclopedia of Systems Biology. Springer, New York, NY. https://doi.org/10.1007/978-1-4419-9863-7_1215

Ranganathan, P., Pramesh, C. S., & Buyse, M. (2016). Common pitfalls in statistical analysis: The perils of multiple testing. *Perspect Clin Res*. 2016 Apr-Jun;7(2):106-7. doi: 10.4103/2229-3485.179436. PMID: 27141478; PMCID: PMC4840791.

VanderWeele, T. J.,  & Mathur, M. B. Some desirable properties of the bonferroni correction: is the bonferroni correction really so bad?, *American Journal of Epidemiology*, Volume 188, Issue 3, March 2019, Pages 617–618, <https://doi.org/10.1093/aje/kwy250>

White, I. R., Royston, P. & Wood, A. M. (2011), Multiple imputation using chained equations: Issues and guidance for practice. Statist. Med., 30: 377-399. <https://doi.org/10.1002/sim.4067>
